# Elucidating multifaceted targets of Marein in cerebral ischemia-reperfusion injury

**DOI:** 10.3389/fchem.2025.1651873

**Published:** 2025-10-09

**Authors:** Weidan Luo, Jian Wang, Ying Fan, Meng Yuan, Wanying Xie, Yang Su, Xingchun Wang, Yi Zhong, Yibo Zhang, Jiaxin Zhan, Xuan Mao, Xinyao Huang, Junxi Long, Xinrui Wang, Tingting Tang, Xingxia Wang

**Affiliations:** 1 Department of Neurology, The Affiliated Hospital, Southwest Medical University, Luzhou, Sichuan, China; 2 Department of General Surgery (Vascular Surgery), The Affiliated Hospital, Southwest Medical University, Luzhou, Sichuan, China; 3 Department of Comprehensive (VIP) Inpatient Ward, Sichuan Clinical Research Center for Cancer, Sichuan Cancer Hospital & Institute, Sichuan Cancer Center, University of Electronic Science and Technology of China, Chengdu, China; 4 Department of Oncology, Luzhou People’s Hospital, Luzhou, Sichuan, China; 5 The Affiliated Stomatological Hospital of Southwest Medical University, Southwest Medical University, Luzhou, Sichuan, China

**Keywords:** Marein, network pharmacology, cerebral ischemia-reperfusion injury, molecular docking, molecular dynamics simulation

## Abstract

**Background:**

Cerebral ischemia-reperfusion injury (CIRI) is a major pathological event in stroke, leading to neuronal damage and neuroinflammation. Marein, a flavonoid derived from *Coreopsis tinctoria*, has demonstrated potential neuroprotective effects, yet its precise mechanisms in CIRI remain unclear.

**Methods:**

Marein-related targets were predicted using SwissTargetPrediction, and cerebral ischemia–reperfusion injury (CIRI)-related targets were obtained from GeneCards. Common targets were identified by Venn analysis, followed by protein–protein interaction network construction and GO/KEGG enrichment analysis. Molecular docking and 100-ns molecular dynamics simulations evaluated interactions between Marein/Edaravone and key targets (PTGS2, SRC, EGFR). *In vitro*, an oxygen–glucose deprivation/reperfusion model in HT22 cells was used to assess cell viability, reactive oxygen species production, and protein expression.

**Results:**

64 common targets (including PTGS2, SRC, EGFR) were identified. Enrichment analyses highlighted involvement in calcium signaling, inflammatory responses. Molecular docking and dynamics confirmed stable binding of Marein to PTGS2, SRC, and EGFR, with favorable binding free energies. *In vitro*, Marein (20 μM) improved viability of OGD/R-exposed HT22 cells, reduced ROS accumulation, and downregulated PTGS2 and SRC expression.

**Conclusion:**

Marein exerts neuroprotective effects against CIRI by targeting PTGS2, SRC, and EGFR, modulating inflammatory and oxidative stress pathways. This study provides a mechanistic basis for Marein’s potential in CIRI therapy.

## 1 Introduction

Stroke, falling under common neurological disorders, has witnessed a climbing incidence rate on a global scale. Accounting for 80%–85% of stroke cases (Cai et al., 2024), ischemic stroke is characterized by elevated morbidity, disability rates, and mortality, necessitating urgent therapeutic advancements in neurological medicine (Shang et al., 2025; Zhang Y. et al., 2024). Cerebral ischemia-reperfusion injury (CIRI) denotes paradoxical exacerbation of cerebral tissue damage following blood flow restoration through thrombolysis or endovascular interventions after transient ischemia (Kumar and Singh, 2024; Lan et al., 2024). CIRI can lead to a series of severe brain diseases as well as varying degrees of disability or death (Lan et al., 2024; Yuan et al., 2021). This secondary injury cascade precipitates hemorrhagic transformation, neurological deterioration, cerebral edema, and progressive infarction, significantly compromising clinical outcomes (Kumar and Singh, 2024). Current therapeutic strategies—including nimodipine administration, Edaravone-based pharmacotherapy, and mechanical thrombectomy—demonstrate limited efficacy in mitigating CIRI progression (Liu et al., 2025; Zhang C. et al., 2024; Zheng et al., 2025). Studies have shown that, stroke as the predominant contributor to premature mortality in China, with ischemic stroke exhibiting incremental incidence rates, high recurrence, and persistent mortality despite gradual prevalence increases (Jiang et al., 2025; Liu et al., 2024; Zhao et al., 2023). These trends underscore the imperative for optimized CIRI management strategies in global healthcare systems (Li Z. et al., 2024; Mandalaneni et al., 2025).

The pathophysiological complexity of CIRI involves multilevel interactions among energy metabolism dysregulation, oxidative stress, neuroinflammation, excitotoxic cascades, and apoptotic signaling (Cai et al., 2024; Zheng H. et al., 2024). Cerebral tissue, exhibiting high oxygen dependence for glucose aerobic metabolism, undergoes rapid ATP depletion during ischemia. Subsequent mitochondrial respiratory chain dysfunction triggers anaerobic glycolysis, lactate accumulation, and intracellular acidosis, culminating in ionic pump failure, cytotoxic edema, and reversible neuronal impairment (Hwang et al., 2024; Yang et al., 2025). Reperfusion paradoxically amplifies injury through the increase of free radicals, calcium dyshomeostasis, and inflammatory hyperactivation. These processes synergistically disrupt blood-brain barrier integrity, potentiate neural apoptosis, and exacerbate cerebral lesions (Wu et al., 2010; Yuan et al., 2021).

Marein (C_21_H_22_O_11_), a flavonoid glucoside isolated from *Coreopsis tinctoria* capitulum, demonstrates multimodal bioactivity relevant to CIRI pathophysiology (Jiang et al., 2016a; Li Y. et al., 2022). Structural analysis reveals a characteristic flavonoid skeleton conjugated with a glucose moiety, facilitating its pharmacokinetic properties (Li Y. et al., 2022). Marein possesses a variety of biological activities. The reported biological activities of Marein so far include exerting antioxidant and anti-inflammatory activities by inhibiting the reactive oxygen species (ROS)/nuclear factor κB (NF-κB) pathway and activating the nuclear factor erythroid 2-related factor 2 (Nrf2)/ARE pathway; exerting neuroprotective activities by reducing damage to mitochondrial function and activating the AMPK signaling pathway (Jiang et al., 2016b); improving insulin resistance activity induced by high glucose in HepG2 cells by promoting glucose uptake, increasing glycogen synthesis, and reducing gluconeogenesis (Jiang et al., 2016a); as well as exhibiting anti-inflammatory activities by inhibiting LPS-induced NF-κB activation to reduce the number of osteoclasts and down-regulating the expression of pro-inflammatory cytokines (Li Y. et al., 2022).

Notably, existing studies have demonstrated that regulating lipid metabolism can improve neurological function (Jiang et al., 2025; Zheng et al., 2025). Comparative phytopharmacological studies highlight structural analogs like isoliquiritigenin—a licorice-derived flavonoid—exert neuroprotection via Nrf2-mediated oxidative stress mitigation and mitochondrial stabilization (Lan et al., 2024; Zhang H. et al., 2024); Similarly, baicalein attenuates cerebral reperfusion injury through SIRT6-dependent ferroptosis inhibition and calpain1/AIF pathway modulation (Wu et al., 2010; Li S. et al., 2022; Z et al., 2025); These findings corroborate the therapeutic potential of flavonoid compounds in CIRI management via antioxidant, anti-apoptotic, and anti-inflammatory mechanisms (Zhang et al., 2006). Given Marein’s structural congruence and demonstrated bioactivity profile, we hypothesize its capacity to target multiple CIRI pathways, warranting systematic investigation into its cerebroprotective mechanisms.

Intracellular calcium ions serve as critical messengers in neuronal signal transduction within the brain. The foundation for neuronal survival is made up of calcium ions, the plasma membrane, mitochondria, and endoplasmic reticulum. Calcium homeostasis imbalance during CIRI may result in intracellular calcium overload. The brain’s reliance on glucose for energy during the ischemic phase raises the intracellular calcium load even more, as glucose metabolism disorders under ischemia disrupt the normal function of ionic pumps involved in calcium transport (Han et al., 2022). Excessive intracellular calcium ions cause cell death by activating calcium-dependent enzymes, including calpains and Ca^2+^/calmodulin-dependent protein kinases (CaMK). Oxidative stress is another key driver of neuronal death, and high mitochondrial matrix Ca^2+^ induces respiratory chain dysfunction, which promotes excessive ROS production and ultimately oxidative stress (Kalogeris et al., 2014).

**FIGURE 1:**
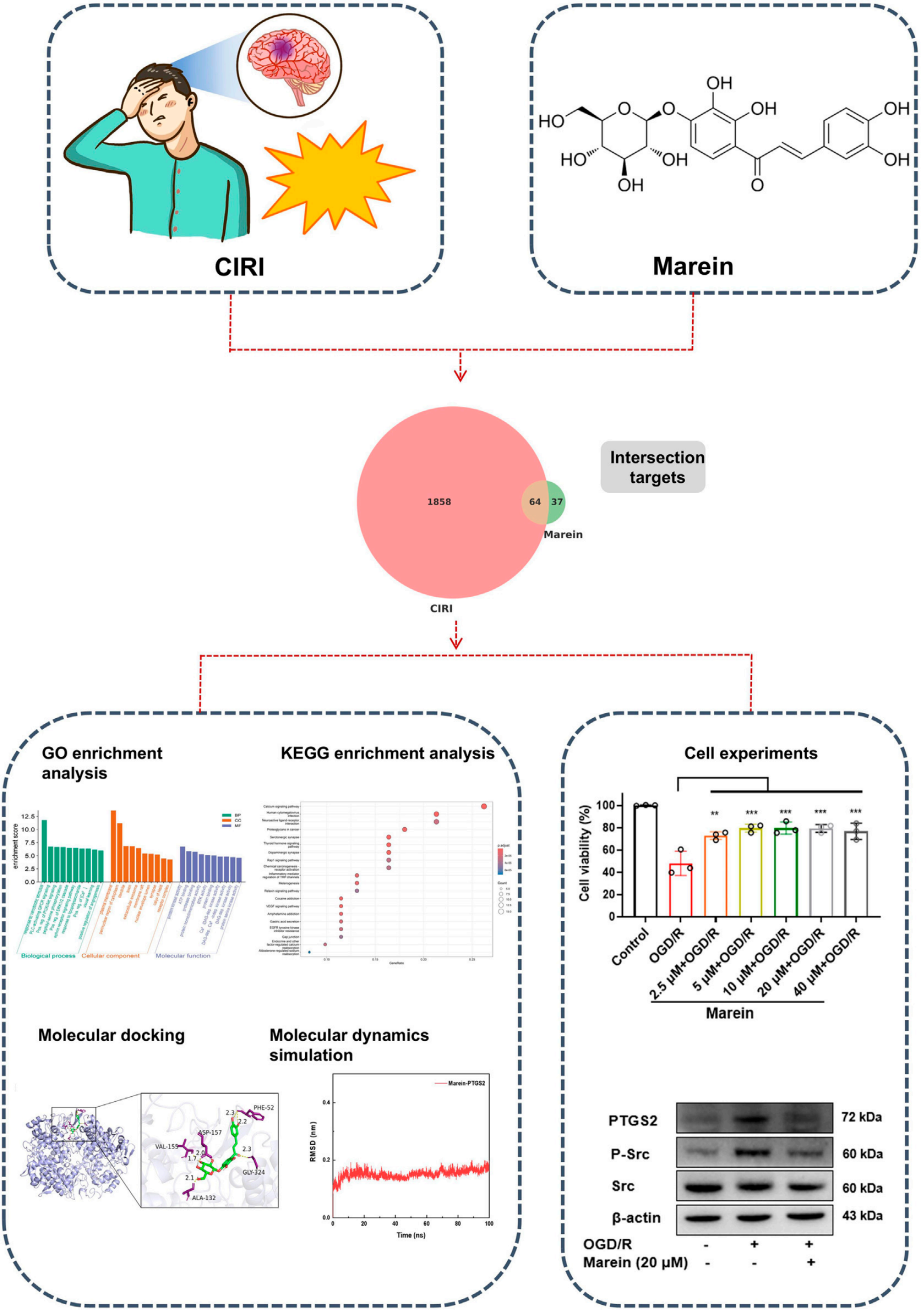
The mechanisms of Marein in anti- CIRI.

This study integrates network pharmacology, molecular docking, molecular dynamics simulations and experimental validation to explore the potential mechanisms of Marein in treating CIRI from multiple perspectives (Figure 1). Network pharmacology, which blends pharmacology with information networks, has been used extensively and draws on systems biology and bioinformatics. It filters drug and disease targets from large datasets and predicts the multifaceted mechanisms of drug action (Zhou et al., 2020). Based on molecular structures, molecular docking helps anticipate interactions between molecules and biological targets, calculate binding free energy, and describe structure-activity connections by extracting valuable information from the 3D structures of targets (Pinzi and Rastelli, 2019). Building upon previous research, this study incorporates molecular dynamics simulation techniques to investigate the conformational changes in proteins induced by ligand binding or unbinding, as well as the binding stability between proteins and molecules (Wu et al., 2022).

This work attempts to uncover possible targets and pathways by examining the molecular processes of Marein in CIRI, hence exposing the therapeutic potential of Marein against CIRI. In addition to offering insightful information for upcoming clinical applications, this study theoretically supports innovative therapy approaches for CIRI.

## 2 Materials and methods

### 2.1 Obtaining the target of Marein

The CAS number of Marein (CAS 535-96-6) was input into PubChem, and the SMILES of Marein was acquired. Then the SMILES number was input into the SwissTarget Prediction database (Luo et al., 2023), with the species limited to *Homo sapiens.* A probability threshold >0 was applied. The target file of Marein was obtained after retrieval and removal of duplicate values.

### 2.2 Obtaining the targets related to CIRI

To collect gene targets associated with CIRI, we performed a retrieval in the Genecards database using “Cerebral ischemia-reperfusion injury” as the key search term (Luo et al., 2023). A relevance score threshold of >0 was applied to filter the results based on correlation coefficients. Following the retrieval, duplicate entries were removed to generate a final set of CIRI-related gene targets.

### 2.3 Construction of the Marein target-disease network

The potential targets of Marein were intersected with the targets associated with cerebral CIRI. The intersection results were visualized using Cytoscape 3.10.2 to generate a Venn diagram, and the overlapping targets were considered the potential therapeutic targets of Marein against CIRI (Wang et al., 2024). These common targets were then imported into the STRING database (version 12.0, https://string-db.org/), with the species restricted to *Homo sapiens* and the minimum required interaction score set to 0.4 (medium confidence), to construct a protein–protein interaction (PPI) network (Luo et al., 2023). The resulting interaction data were exported and further visualized using Cytoscape 3.10.2. The overlapping targets were arranged in a circular layout according to their degree values in descending order, with colors transitioning continuously from red to blue corresponding to the degree values. The three targets with the highest degree values, positioned at the center with the most intense red coloration and the largest node sizes, were identified as hub genes.

### 2.4 GO and KEGG pathway enrichment analysis

The core targets were imported into the DAVID database for functional enrichment analysis (Li X. et al., 2022; Luo et al., 2023; Wang et al., 2024). Gene Ontology (GO) analysis was performed and categorized into Biological Process (BP), Cellular Component (CC), and Molecular Function (MF) entries. BP entries included processes such as protein phosphorylation, intracellular signal transduction, and regulation of gene expression; CC entries encompassed cellular structures such as the presynaptic membrane, plasma membrane, and γ-secretory complex; MF entries involved molecular activities such as protein kinase activity and ATP binding. For visualization purposes, the top 10 GO terms in each category (BP, CC, and MF) and the top 20 KEGG pathways were selected to generate the corresponding enrichment graphs. KEGG pathway results were further converted into a bubble chart using R. Although these figures display the top-ranked entries, the subsequent interpretation focused on those terms and pathways most relevant to stroke pathology.

### 2.5 Molecular docking validation of Marein with disease-related target proteins

The 3D structures of Marein and Edaravone were downloaded from the PubChem database (https://pubchem.ncbi.nlm.nih.gov/). Using AutoDockTools-1.5.7 software (Goodsell et al., 1996; Morris et al., 1996; Morris et al., 2009), the Marein molecule was preprocessed by adding hydrogen atoms and designated as the ligand. The preprocessed ligand data were saved in PDBQT format. Based on the intersection of drug targets and disease-related targets, three proteins—Prostaglandin-Endoperoxide Synthase 2 (PTGS2, also known as Cyclooxygenase-2; PDB ID: 5F1A), SRC (PDB ID: 1A09), and EGFR (PDB ID: 1M14)—were selected for docking. The 3D structures of these proteins were downloaded from the Protein Data Bank (https://www.rcsb.org/) and imported into AutoDockTools for preprocessing, including removal of water molecules and addition of hydrogen atoms. The proteins were then designated as receptors and saved in PDBQT format.

For docking, the preprocessed protein and ligand files (Marein and Edaravone) were imported into AutoDockTools to establish the Grid Box. Known inhibitors—celecoxib for PTGS2, imatinib for SRC, and gefitinib for EGFR—were docked as controls to validate that the grid parameters accurately captured the active sites. The grid box dimensions were set to encompass the entire active site region with sufficient padding (typically 60 Å × 60 Å × 60 Å with a grid spacing of 0.375 Å). After sequentially docking Marein and then Edaravone, the docking calculations were performed. Finally, docking results were imported into PyMOL software (Luo et al., 2023; Zou et al., 2023) for visualization and analysis of ligand–protein interactions. This approach ensures that the selected active sites are properly justified based on both structural data and published literature, providing a reliable foundation for the molecular docking studies.

### 2.6 Molecular dynamics simulations

Molecular dynamics simulations were performed on the protein - ligand complex using the GROMACS 2020.3 software (Zheng Z. et al., 2024) to probe the interactions between the receptor and the ligand. By utilizing the amber99sb-ildn force field and the General Amber Force Field (GAFF), the parameters and topological structures of the protein and the ligand were separately produced. The size of the simulation box was configured to guarantee that the distance from every atom of the protein to the box exceeded 1.0 nm. The box was populated with SPC216 water molecules, and the water molecules were substituted with Na^+^ and Cl^−^ ions to render the simulation system electrically neutral. The steepest descent method was employed to optimize the entire system, thereby reducing the unreasonable contacts or atomic overlaps within the system. To achieve the sufficient pre-equilibration of the simulation system, simulations of the NVT ensemble and the NPT ensemble were conducted for 100 picoseconds (ps) at 300 K (K) and 1 bar respectively. Subsequently, a molecular dynamics simulation lasting for 100 nanoseconds (ns)was carried out under periodic boundary conditions. Employing the V-rescale and Parrinello-Rahman techniques, the temperature (300 K) and pressure (1 bar) were regulated with an integration time of 2 femtoseconds (fs). Subsequently, the Particle Mesh Ewald (PME) technique was used to calculate long-range electrostatic interactions. The Fourier spacing was set to 0.16 nm (nm), and the LINCS method was utilized to limit all bond lengths. VMD 1.9.3 (Bo et al., 2025) and PyMOL 2.4.1 were employed for visualizing, analyzing, and animating the trajectories (Muheyuddeen et al., 2024). The binding free energy of the complex was calculated by using gmx_mmpbsa (http://jerkwin.github.io/gmxtools) (Pirojsirikul et al., 2024).

### 2.7 Cell culture

HT22 cells (BNCC358041) were obtained from BeNa Culture Collection (Beijing, China). Cells were cultured in high-glucose Dulbecco’s Modified Eagle Medium (DMEM; Gibco, United States) supplemented with 10% fetal bovine serum (FBS; Gibco, United States) and 1% penicillin-streptomycin (Solarbio, China). Cultures were maintained in a humidified incubator at 37 °C with 5% CO_2_. Cells in the logarithmic growth phase were used for all subsequent experiments.

### 2.8 CCK - 8 assay

Cell viability was assessed using the Cell Counting Kit-8 (CCK-8; Hanheng Biotechnology, HB-CCK-500T, Shanghai, China). HT22 cells were seeded into 96-well plates at a density of 5 × 10^4^ cells/mL, 100 μL per well, and incubated overnight at 37 °C with 5% CO_2_ to allow for attachment. For the dose-response experiment under normoxic conditions, cells were treated with 2.5, 5, 10, 20, 40, or 80 μM Marein (Shanghai Taoshu Biotechnology) for 24 h. After treatment, 10 μL of CCK-8 reagent was added directly to each well, and cells were incubated at 37 °C for 1.5 h. Absorbance was measured at 450 nm using a microplate reader (BioTek Instruments, United States). Wells containing only medium and CCK-8 (without cells) served as the blank, and untreated cells were used as the control. For the OGD/R experiments, cells were pretreated with 2.5–40 μM Marein for 24 h, then subjected to oxygen-glucose deprivation by replacing the medium with glucose-free DMEM (Gibco, United States), followed by incubation in a hypoxia chamber (95% N_2_/5% CO_2_) at 37 °C for 8 h. Cells were then reoxygenated in complete medium for 16 h under normoxic conditions (5% CO_2_ at 37 °C). Marein was added only during the reoxygenation period. After reoxygenation, the CCK-8 assay was performed as described above. Each group was tested in triplicate wells, and experiments were repeated independently at least three times.

### 2.9 Intracellular ROS detection

Intracellular ROS levels were measured using the fluorescent probe DCFH-DA (2′, 7′-dichlorodihydrofluorescein diacetate), purchased from Beyotime Biotechnology (Shanghai, China). HT22 cells were seeded into 6-well plates at a density of 5 × 10^5^ cells/well and cultured overnight at 37 °C in a 5% CO_2_ incubator. Cells were pretreated with Marein at concentrations of 5, 10, or 20 μM for 24 h, followed by OGD/R as described in Section 2.8. After reoxygenation, cells were incubated with 10 μM DCFH-DA (diluted in serum-free DMEM) at 37 °C for 40 min in the dark. Following staining, cells were washed three times with phosphate-buffered saline (PBS) to remove excess probe. Fluorescence intensity was detected using a microplate reader (excitation at 488 nm, emission at 525 nm). Untreated cells served as the control, OGD/R cells without Marein as the model group, and wells without DCFH-DA staining were used as the blank. All assays were performed in biological triplicates.

## 3 Results

### 3.1 Target prediction and screening results of Marein

**FIGURE 2:**
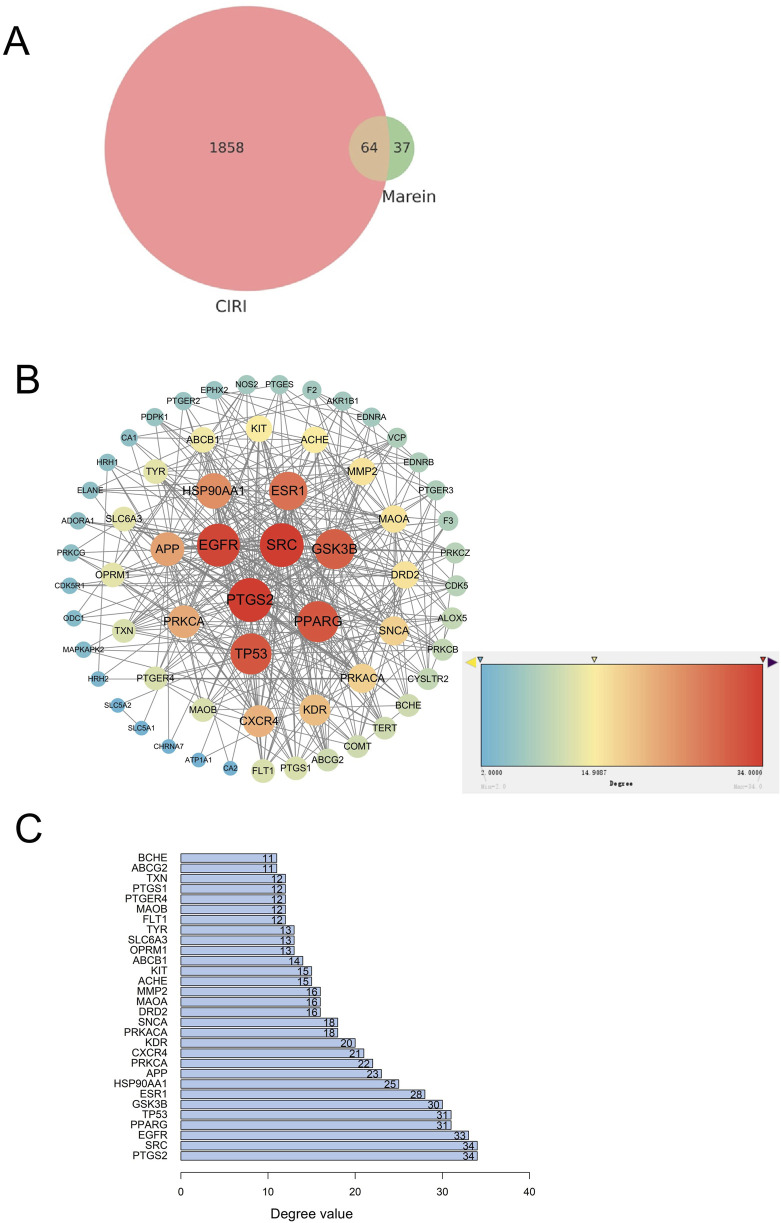
**(A)** Venn diagram showing the distribution of target genes. A total of 1858 CIRI-related targets, 37 Marein-related targets, and 64 overlapping targets were identified. The circle sizes are proportional to the number of genes. **(B)** PPI network of the 64 overlapping targets constructed using the STRING database and visualized in Cytoscape. Node size is scaled according to the degree value, highlighting the hub proteins within the network. **(C)** Bar plot of the degree values of the overlapping targets derived from Cytoscape analysis. The X-axis represents the degree value, and the Y-axis shows the corresponding target genes.

The SMILES identifier for Marein was retrieved from the PubChem database, and 101 potential target genes were identified using SwissTargetPrediction. Additionally, 1,922 target genes associated with CIRI were obtained from the GeneCards database. A Venn diagram (generated via R) identified 64 common genes (e.g., PTGS2, SRC, EGFR, PPARG, TP53, GSK3β, ESR1) as potential therapeutic targets of Marein in CIRI treatment (Figure 2A).

### 3.2 Results of Protein-Protein Interaction network analysis

We constructed a PPI network of the overlapping targets using the STRING database and further analyzed it in Cytoscape (Figure 2B). The network revealed a highly interconnected architecture, in which several nodes exhibited significantly higher degree values, suggesting that these proteins may play pivotal roles in mediating the therapeutic effects of Marein. The PPI network consisted of 30 nodes and 219 edges, with an average node degree of 14.6 and an average local clustering coefficient of 0.697. Edges represented protein interaction strength, with more connections indicating stronger relationships. Core targets (ranked by degree centrality) included PTGS2, SRC, EGFR, PPARG, and TP53 (Figure 2C).

### 3.3 GO enrichment and KEGG pathway analysis

**FIGURE 3:**
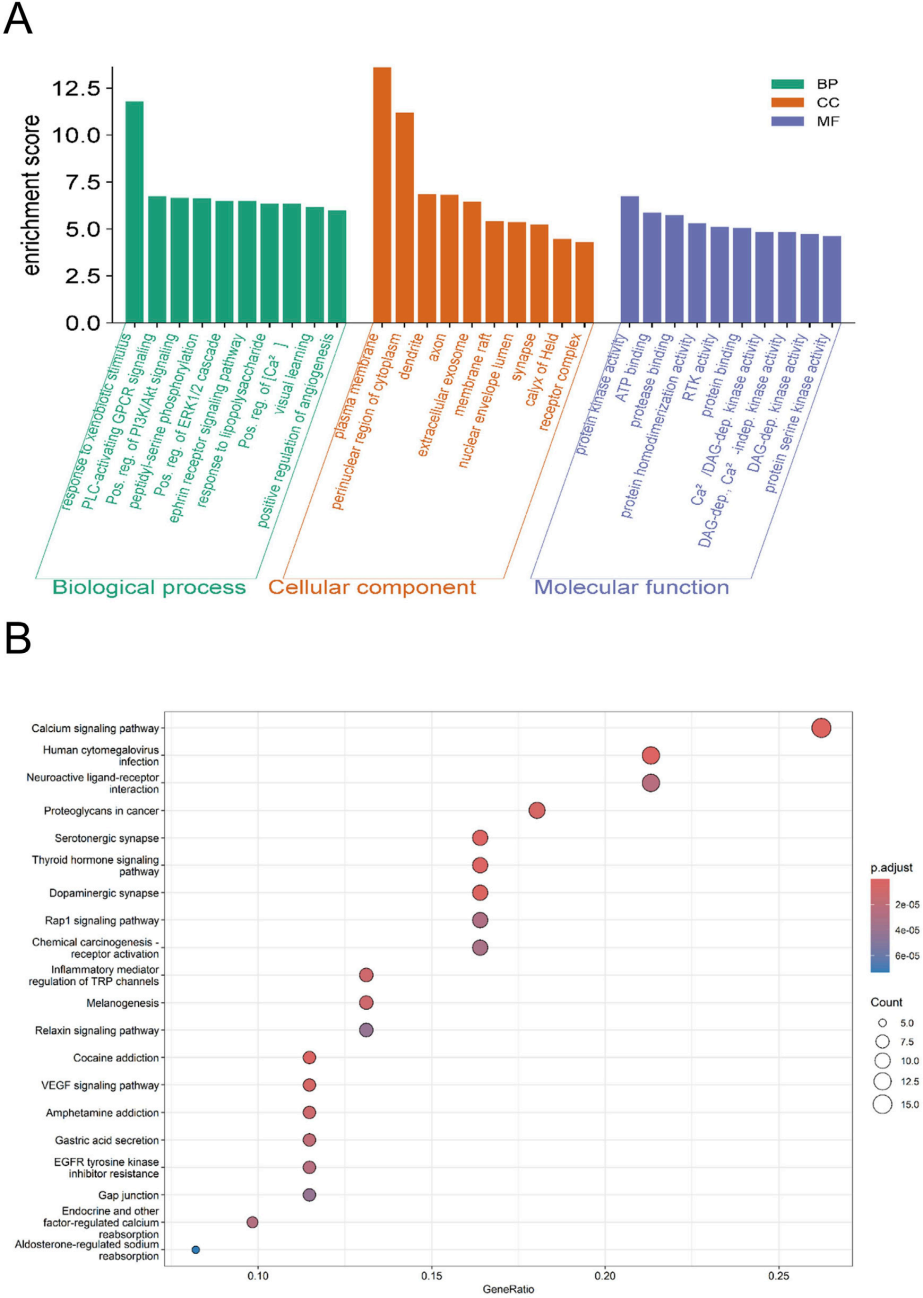
**(A)** Bar chart of GO enrichment results. The bar chart of GO enrichment analysis for the targets of Marein in CIRI includes three dimensions: Biological Process (BP, green), Cellular Component (CC, orange), and Molecular Function (MF, purple). The horizontal axis represents the terms, while the vertical axis represents the enrichment score, reflecting the degree of enrichment for each GO category. **(B)** Bubble chart of KEGG enrichment analysis. The bubble chart of KEGG enrichment analysis features the gene ratio on the horizontal axis and the enriched KEGG pathways on the vertical axis. The color of the bubbles represents the adjusted p-value, with a gradient from red to blue indicating the level of significance; the redder the bubble, the higher the significance, and the bluer the bubble, the lower the significance. The size of the bubbles corresponds to the number of enriched genes, with larger bubbles denoting a greater quantity of genes.

GO enrichment analysis (Figure 3A) revealed that several BP entries are directly implicated in stroke-related mechanisms, including positive regulation of PI3K/AKT signal transduction, positive regulation of ERK1/2 cascade, response to lipopolysaccharide, positive regulation of cytosolic calcium ion concentration, and positive regulation of angiogenesis, reflecting neuronal survival, neuroinflammation, calcium homeostasis, and post-stroke angiogenesis. In the CC category, components such as dendrite, axon, synapse, and receptor complex highlight neural structures susceptible to ischemic injury. For MF entries, protein kinase activity, protein serine kinase activity, ATP binding, and transmembrane receptor protein tyrosine kinase activity indicate involvement in signal transduction and energy metabolism critical for ischemic neurons.

KEGG pathway analysis (Figure 3B) showed multiple pathways relevant to stroke, including Calcium signaling, VEGF signaling, Inflammatory mediator regulation of TRP channels, Neuroactive ligand-receptor interaction, and Rap1 signaling. These pathways collectively cover calcium homeostasis, neuroprotection, angiogenesis, and neuroinflammation, which are central mechanisms in ischemic stroke pathophysiology. Other pathways within the top 20 provide additional context but are less directly associated with stroke. Overall, although the figures present the top-ranked entries, the biological interpretation emphasizes stroke-relevant mechanisms, ensuring meaningful insight into the potential roles of the identified targets.

### 3.4 Molecular docking results of Marein and Edaravone with CIRI-Related target proteins

**FIGURE 4:**
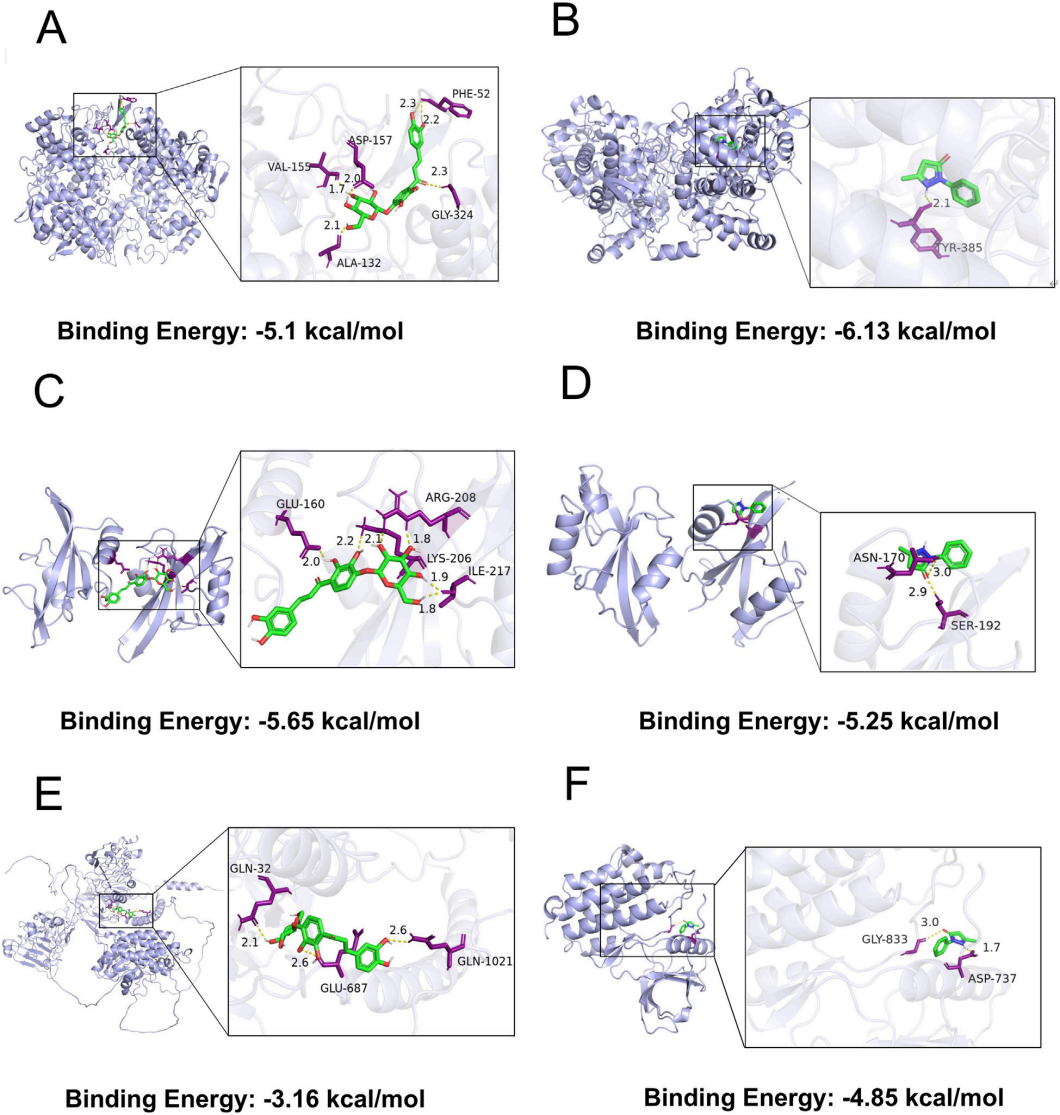
Molecular docking of compounds with target proteins in CIRI. **(A)** Marein-PTGS2. **(B)** Edaravone-PTGS2. **(C)** Marein-Src. **(D)** Edaravone-Src. **(E)** Marein-EGFR. **(F)** Edaravone-EGFR.

Molecular docking was performed to investigate the binding interactions between Marein or Edaravone and three key proteins implicated in CIRI: PTGS2, SRC, and EGFR. The binding affinities, expressed as minimum binding energies, along with residue-specific interactions, are summarized as follows. Marein demonstrated a binding energy of −5.1 kcal/mol with PTGS2. The molecule was accommodated within the substrate-binding pocket. These interactions, localized within the catalytic cavity, suggest a stable association that could potentially interfere with the enzymatic activity of PTGS2 (Figure 4A). The binding affinity between Marein and SRC was stronger, with a minimum binding energy of −5.65 kcal/mol. Marein targeted the kinase domain, engaging in hydrogen bonding residues integral to SRC’s kinase function. This multi-point interaction pattern indicates a potential inhibitory effect on SRC-mediated signaling (Figure 4C). Marein exhibited a comparatively weaker interaction with EGFR (−3.16 kcal/mol). Nonetheless, it formed hydrogen bonds within the tyrosine kinase domain. Though binding affinity was lower than that observed for PTGS2 and SRC, the positioning of Marein within the ATP-binding pocket suggests a possible modulatory effect on EGFR activation (Figure 4E).

Edaravone displayed a high affinity for PTGS2, with a binding energy of −6.13 kcal/mol. Notably, a key hydrogen bond was formed with TYR-385, a residue located in the substrate-recognition region, implying that Edaravone may competitively inhibit substrate binding and consequently modulate PTGS2 enzymatic activity (Figure 4B). The minimum binding energy for Edaravone with SRC was −5.25 kcal/mol. Edaravone engaged residues ASN-170 and SER-192, which belong to the ATP-binding motif, indicating that its binding may interfere with SRC phosphorylation and downstream signaling (Figure 4D). As depicted in Figure 4F, edaravone interacted with EGFR, forming favorable contacts with key residues, namely ASP ‐ 737 and GLY ‐ 833. The calculated binding energy was - 4.85 kcal/mol, indicating a spontaneous and energetically favorable binding process between edaravone and EGFR. The docking results implied that edaravone could bind to EGFR with a certain degree of affinity.

Marein exhibited the strongest binding affinity to SRC (−5.65 kcal/mol), implying a significant capacity to modulate SRC-driven pathways in CIRI, likely through inhibition of kinase activity. Its relatively lower affinity for EGFR indicates a lesser, though still relevant, influence on EGFR-mediated signaling. Conversely, Edaravone’s highest affinity was observed with PTGS2 (−6.13 kcal/mol), suggesting its primary role in regulating PTGS2-associated inflammatory responses. Its moderate affinity to SRC may contribute to additional modulatory effects. To further validate these docking predictions, molecular dynamics simulations were conducted focusing on PTGS2 and SRC, as these targets showed the most prominent binding characteristics with the respective ligands. These simulations aimed to assess the stability and dynamics of the complexes, providing insight into the sustained interactions and potential allosteric effects. In conclusion, the molecular docking results reveal distinct binding patterns of Marein and Edaravone with CIRI-associated proteins. The identified residue-level interactions, particularly within critical functional domains, provide a mechanistic basis for their pharmacological actions. When integrated with molecular dynamics findings, these data will enhance the understanding of how these compounds may modulate molecular pathways implicated in CIRI.

### 3.5 Molecular dynamics simulation

**FIGURE 5:**
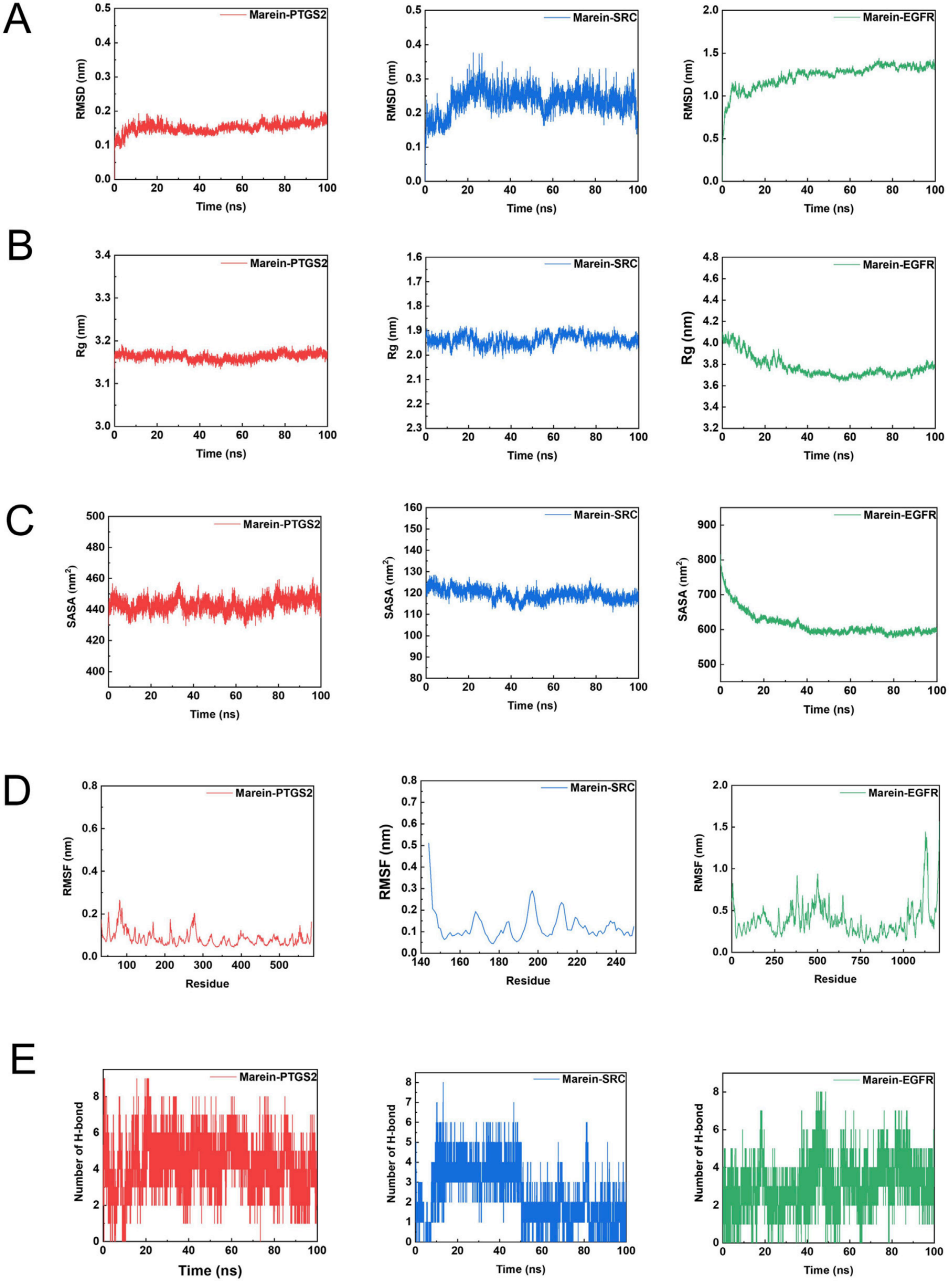
Key parameters from molecular dynamics simulations of Marein - protein complexes. **(A)** Root Mean Square Deviation (RMSD) profiles of three complexes (Marein - PTGS2, Marein - SRC, Marein - EGFR) over the simulation time (ns). **(B)** Root Mean Square Fluctuation (RMSF) plots of the three complexes as a function of time (ns). **(C)** Radius of Gyration (Rg) variations of the three complexes with time (ns). **(D)** Distribution of the number of hydrogen bonds in the three complexes during the simulation period (ns). **(E)** Solvent - accessible surface area (SASA) values of the three complexes plotted against time (ns).

**FIGURE 6:**
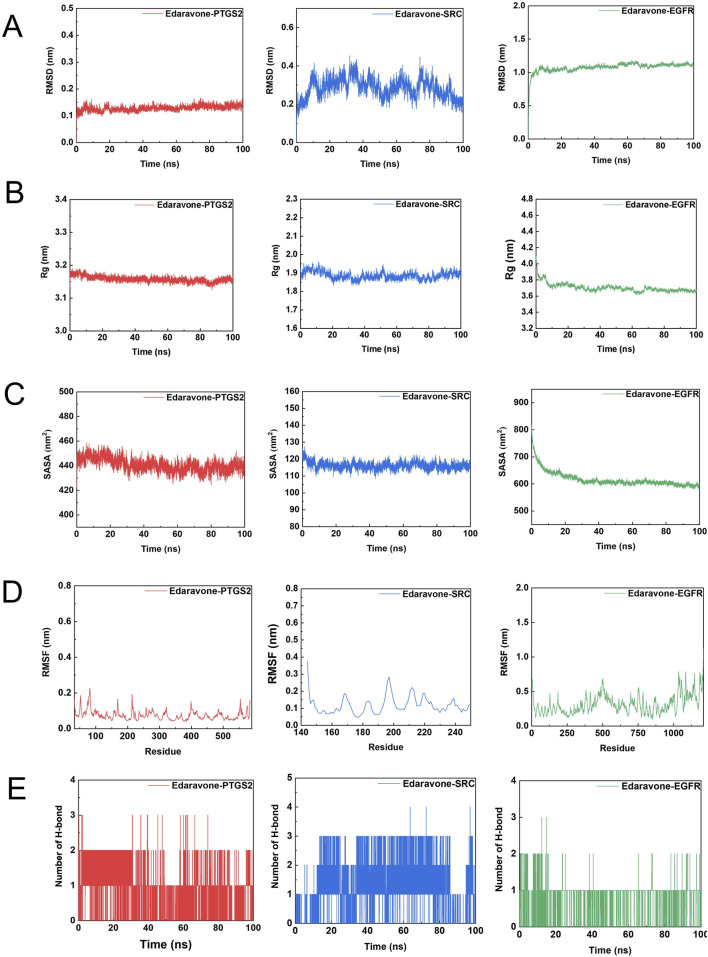
Key parameters from molecular dynamics simulations of Edaravone - protein complexes. **(A)** Root Mean Square Deviation (RMSD) of Edaravone - PTGS2, Edaravone - SRC, and Edaravone - EGFR complexes over time (ns). **(B)** Radius of Gyration (Rg) of the three Edaravone - protein complexes as a function of simulation time (ns). **(C)** Radius of Gyration (Rg) variations of Edaravone - PTGS2, Edaravone - SRC, and Edaravone - EGFR complexes with time (ns). **(D)** Root Mean Square Fluctuation (RMSF) of the three Edaravone - protein complexes plotted against residue number. **(E)** Number of hydrogen bonds in Edaravone - PTGS2, Edaravone - SRC, and Edaravone - EGFR complexes over the simulation time (ns).

The stability and interaction characteristics of the protein–ligand complexes were evaluated through molecular dynamics simulations. As shown in Figures 5A, 6A, the Edaravone–PTGS2, Marein–PTGS2, Edaravone–SRC, Marein–SRC, Edaravone–EGFR, and Marein–EGFR complexes reached equilibrium after approximately 10, 10, 70, 20, 10, and 70 ns of simulation, respectively. The corresponding mean RMSD values were 0.1276 ± 0.0099, 0.1516 ± 0.0151, 0.2833 ± 0.0518, 0.2362 ± 0.0365, 1.08 ± 0.0690, and 1.23 ± 0.1394 nm. All systems exhibited small RMSD fluctuations, indicating structural stability throughout the simulation period. Protein compactness was further assessed by calculating the radius of gyration (Rg) (Figures 5B, 6B). The Rg values of the Edaravone–EGFR and Marein–EGFR complexes displayed a gradual decline over time, while those of the PTGS2 and SRC complexes remained essentially unchanged. The absence of an upward trend in Rg values across all complexes suggests stable protein–ligand binding. Comparable Rg values between the Edaravone and Marein complexes further support the binding stability of Marein. Solvent-accessible surface area (SASA) analysis (Figures 5C, 6C) revealed a decreasing trend in SASA for the Edaravone–EGFR and Marein–EGFR complexes, whereas the PTGS2 and SRC complexes exhibited minimal changes. The lack of any upward shift in SASA values indicates the absence of structural loosening during the simulation.

The flexibility of individual protein regions was examined using root mean square fluctuation (RMSF) analysis (Figures 5D, 6D). The mean RMSF values for the Edaravone–EGFR, Marein–EGFR, Edaravone–PTGS2, Marein–PTGS2, Edaravone–SRC, and Marein–SRC complexes were 0.3171, 0.3981, 0.0752, 0.0847, 0.1183, and 0.1240 nm, respectively—all below 0.35 nm—indicating limited structural fluctuations. The similarity in RMSF values between the Edaravone and Marein complexes suggests that Marein binding does not cause significant conformational alterations. Hydrogen bond analysis (Figures 5E, 6E) showed mean hydrogen bond numbers of 0.912, 3.189, 0.997, 4.273, 1.282, and 2.430 for the Edaravone–EGFR, Marein–EGFR, Edaravone–PTGS2, Marein–PTGS2, Edaravone–SRC, and Marein–SRC complexes, respectively, confirming stable hydrogen-bond-mediated interactions in all systems.

Binding free energy calculations using the gmx_mmpbsa tool (https://jerkwin.github.io/gmxtool/) further quantified the thermodynamic stability of each complex (Tables 1, 2). The calculated binding free energies were −104.421, −61.590, −84.640, −60.417, −36.433, and −36.193 kJ/mol, respectively, all negative, indicating favorable binding. Van der Waals forces were the predominant contributors to the binding affinity. In the EGFR complexes, Edaravone exhibited stronger binding than Marein, suggesting more robust and stable interactions, potentially related to conformational adjustments and altered torsion angles of residues in the binding site.

**TABLE 1:** Molecular Mechanics Poisson-Boltzmann Surface Area (MM-PBSA) analysis of protein-Marein interactions.

Energy	Marein-EGFR	Marein-PTGS2	Marein-SRC
Van der Waals Energy (KJ/mol)	−169.680	−133.183	−71.830
Electrostatic energy (kJ/mol)	−91.900	−125.060	−37.190
Polar solvation energy (KJ/mol)	223.457	220.719	87.609
Nonpolar solvation Energy (KJ/mol)	−23.466	−22.892	−14.782
Total Binding Energy (KJ/mol)	−61.590	−60.417	−36.193

**TABLE 2:** MM-PBSA analysis of protein-Edaravone interactions.

Energy	Edaravone-EGFR	Edaravone-PTGS2	Edaravone-SRC
Van der Waals Energy (KJ/mol)	−134.262	−123.961	−61.323
Electrostatic energy (kJ/mol)	−40.254	−26.262	−40.952
Polar solvation energy (KJ/mol)	85.220	81.096	77.673
Nonpolar solvation Energy (KJ/mol)	−15.125	−15.513	−11.831
Total Binding Energy (KJ/mol)	−104.421	−84.640	−36.433

**FIGURE 7:**
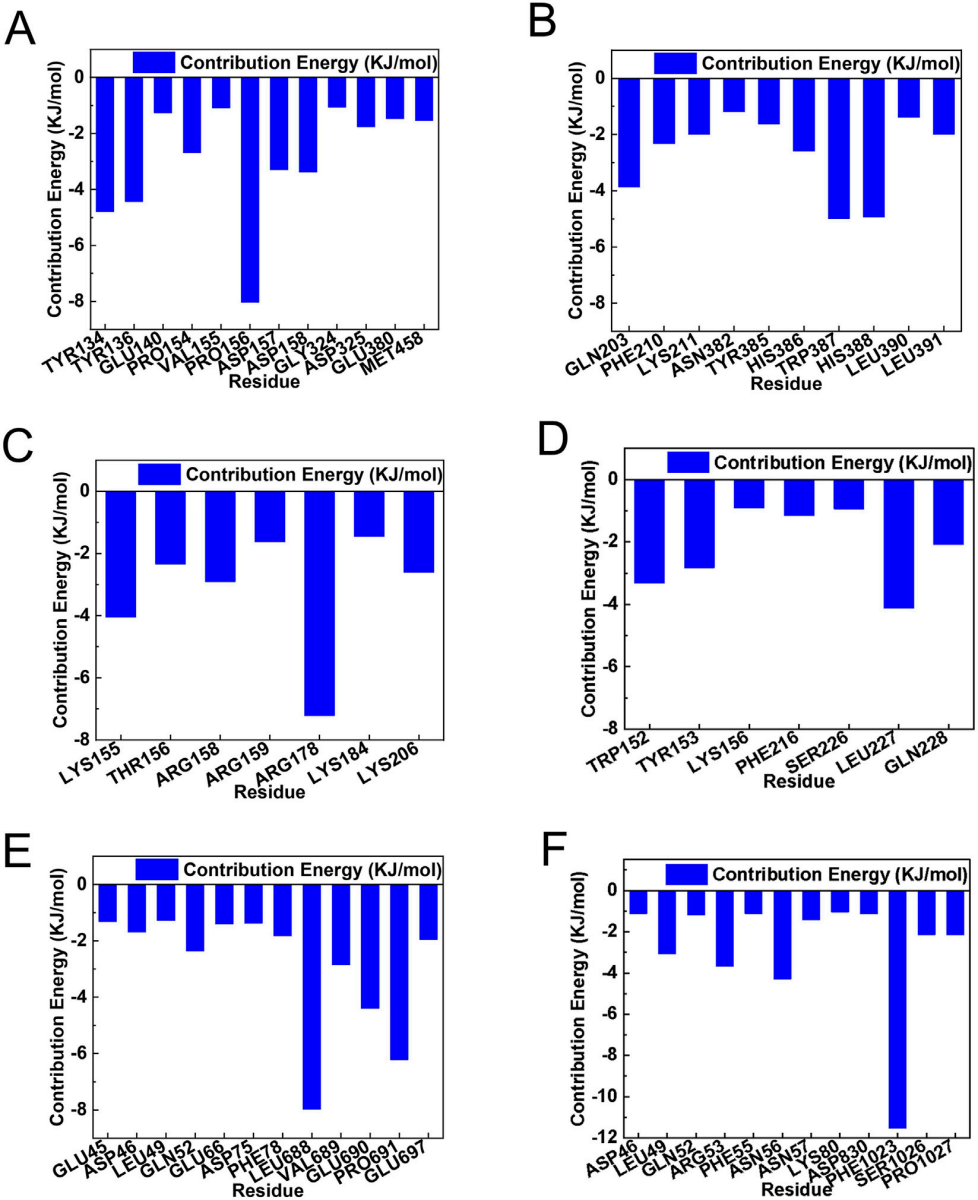
Residue - specific contribution energies from molecular mechanics calculations. **(A)** Contribution energy of each residue in the Marein - PTGS2 complex, with energy values in KJ/mol plotted against residue number. **(B)** Contribution energy of each residue in the Edaravone - PTGS2 complex, with energy values in KJ/mol plotted against residue number. **(C)** Contribution energy of each residue in the Marein - SRC complex, with energy values in KJ/mol plotted against residue number. **(D)** Contribution energy of each residue in the Edaravone - SRC complex, with energy values in KJ/mol plotted against residue number. **(E)** Contribution energy of each residue in the Marein - EGFR complex, with energy values in KJ/mol plotted against residue number. **(F)** Contribution energy of each residue in the Edaravone - EGFR complex, with energy values in KJ/mol plotted against residue number.

Residue-level interaction analysis (Figure 7) identified the principal binding residues for each complex. In PTGS2, Marein interacted primarily with TYR134, TYR136, GLU140, PRO154, VAL155, PRO156, ASP157, ASP158, GLY324, ASP325, GLU380, and MET458, with PRO156 as a key site, while Edaravone bound mainly to GLN203, PHE210, LYS211, ASN382, TYR385, HIS386, TRP387, HIS388, LEU390, and LEU391 (Figures 7A,B). In SRC, Marein interacted with LYS155, THR156, ARG158, ARG159, ARG178, LYS184, and LYS206, whereas Edaravone engaged TRP152, TYR153, LYS156, PHE216, SER226, LEU227, and GLN228 (Figures 7C,D). In EGFR, Marein primarily contacted GLU45, ASP46, LEU49, GLN52, GLU66, ASP75, PHE78, LEU688, VAL689, GLU690, PRO691, and GLU697, while Edaravone interacted with ASP46, LEU49, GLN52, ARG53, PHE55, ASN57, LYS80, ASP830, PHE1023, SER1026, and PRO1027 (Figures 7E,F). These findings provide a detailed structural basis for the observed differences in binding stability and interaction patterns between Marein and the standard drug Edaravone. As shown in Supplementary Figures S1–S6 and Supplementary Video S1 both compounds remained stably bound within the active sites throughout the simulation trajectories, despite time-dependent conformational adjustments of the target proteins. These results highlight the persistent drug–target interactions and conformational adaptability of the binding pockets.

### 3.6 Marein enhances cell viability and attenuates oxidative stress in OGD/R-exposed HT22 cells

**FIGURE 8:**
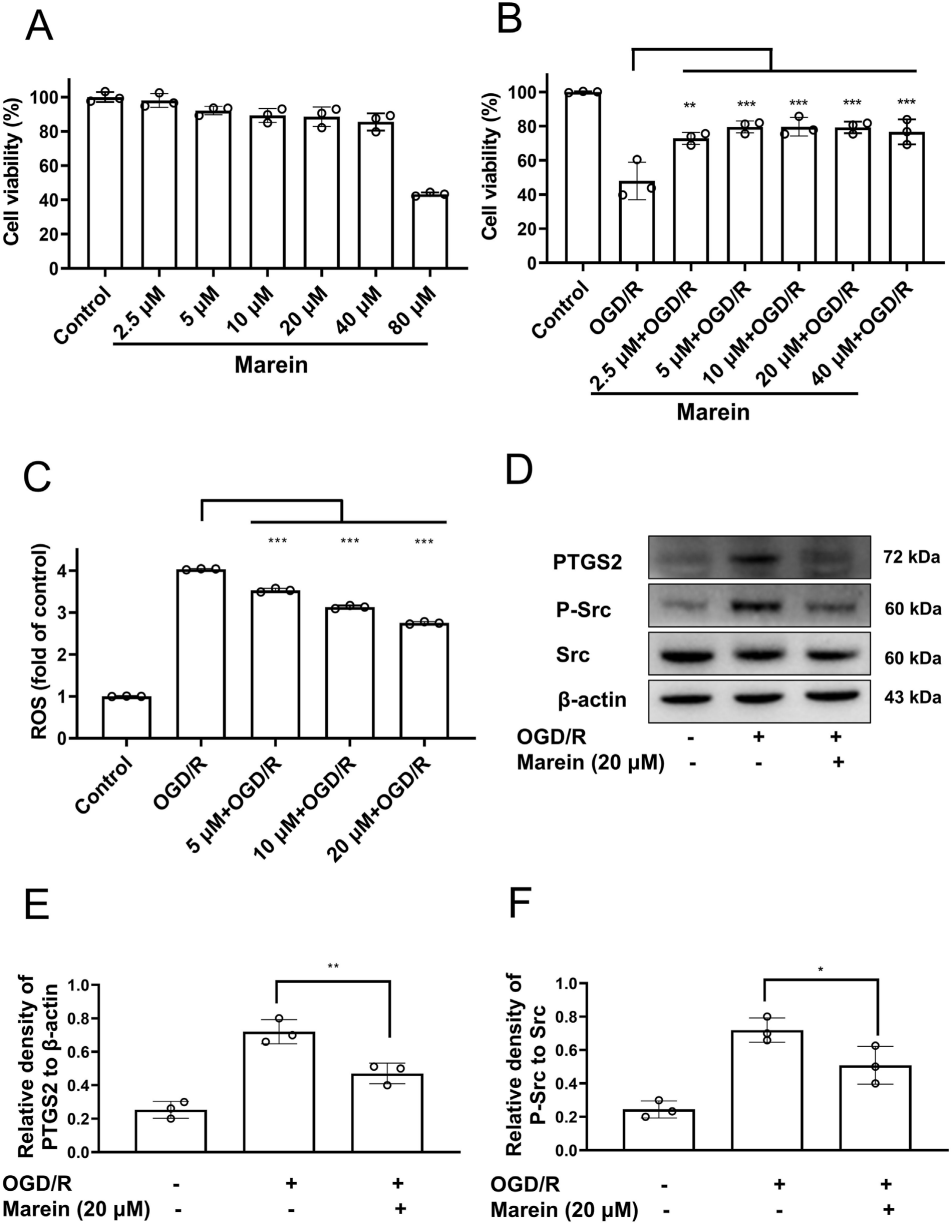
Marein protects HT22 cells against OGD/R injury. **(A)** Cell viability of HT22 cells treated with increasing concentrations of Marein (2.5–80 μM) for 24 h, measured by CCK-8 assay. Data are presented as mean ± SD (n = 3). **(B)** Cell viability of HT22 cells subjected to OGD/R, with or without Marein pretreatment (2.5–40 μM). Viability assessed via CCK-8 assay. **(C)** Intracellular reactive oxygen species (ROS) levels in HT22 cells after OGD/R with or without Marein (5–20 μM) pretreatment, detected by DCFH-DA probe. Data are shown as fold change relative to control. **(D)** Representative Western blots of PTGS2, P - Src, Src, and β - actin membrane Western blots showing protein expression levels in Supplementary Figure S7. **(E)** Quantification of PTGS2 protein levels relative to β - actin. **(F)** Quantification of P - Src protein levels relative to Src. Mean ± SD, n = 3; *p < 0.05, **p < 0.001, ***p < 0.001 vs OGD/R group.

Marein exhibited a concentration-dependent effect on HT22 cell viability (Figure 8A). Treatment with concentrations up to 40 μM for 24 h did not impair viability, maintaining levels above 90% of control, whereas exposure to 80 μM resulted in significant cytotoxicity. Consequently, concentrations ranging from 2.5 to 40 μM were selected for subsequent experiments. Under OGD/R conditions, cell viability dropped to approximately 40%, consistent with severe ischemic insult. Marein pretreatment at 10–40 μM significantly improved survival, restoring viability to 80%–90% (Figure 8B). Furthermore, Marein effectively suppressed OGD/R-induced intracellular ROS accumulation. ROS levels increased nearly fourfold following OGD/R exposure, but Marein at 10–20 μM reduced ROS generation to about threefold of control values (Figure 8C). These findings suggest that Marein confers cytoprotection by both preserving cellular viability and mitigating oxidative stress.

### 3.7 Validation of PTGS2 and SRC expression in OGD/R model

Western blot analysis was performed to determine the expression of PTGS2 and phosphorylated Src (P-Src) in HT22 cells subjected to OGD/R, with or without Marein treatment. As shown in Figure 8D, OGD/R exposure markedly increased PTGS2 and P-Src levels compared with the control group. Quantitative densitometry demonstrated that PTGS2 expression was approximately 2.4-fold higher in the OGD/R group than in the control group (*P* < 0.01; Figure 8E). Likewise, the P-Src/Src ratio increased by nearly 3.1-fold under OGD/R conditions (*P* < 0.05; Figure 8F). Treatment with Marein (20 μM) significantly reduced PTGS2 expression by about 35% (*P* < 0.01) and decreased the P-Src/Src ratio by approximately 30% (*P* < 0.05) compared to the OGD/R group. These data indicate that Marein attenuates the OGD/R-induced upregulation of PTGS2 and Src phosphorylation in HT22 cells. These results support the network pharmacology and molecular docking findings, suggesting that Marein may exert neuroprotective effects by modulating PTGS2- and SRC-mediated pathways.

## 4 Discussion

Ischemic stroke constitutes approximately 87% of cerebrovascular accidents worldwide, representing a leading cause of mortality and disability (Saini et al., 2021). Although timely reperfusion can salvage penumbral tissues, the restoration of blood flow paradoxically triggers secondary tissue damage, clinically defined as CIRI. This pathological cascade arises from intricate interactions among neuroinflammation, oxidative stress, calcium dyshomeostasis, excitotoxicity, mitochondrial impairment, and regulated cell death pathways including apoptosis, autophagy, and ferroptosis. These mechanisms collectively underscore the imperative for multi-target therapeutic strategies against CIRI (Z et al., 2025). Emerging evidence indicates that CIRI activates multiple pathological cascades including programmed cell death, excessive free radical generation, energy metabolism dysfunction, excitatory amino acid toxicity, and aberrant nitric oxide synthesis (Yuan et al., 2023).

Marein is a flavonoid monomer isolated from the capitulum of *Coreopsis tinctoria* (Onorati et al., 2018), demonstrates multi-target therapeutic potential against CIRI through network pharmacology predictions, molecular docking analyses, and molecular dynamics simulations. Integrated analyses encompassing intersection screening and GO functional annotation identified key targets of Marein: PTGS2, and SRC. These targets are critically involved in inflammatory responses, oxidative stress modulation, and apoptotic regulation (Roskoski, 2015). For instance, EGFR and SRC serve as pivotal regulators of cell survival and inflammatory signaling, while PTGS2 acts as the rate-limiting enzyme in prostaglandin biosynthesis (Martín-Vázquez et al., 2023; Talukdar et al., 2020).

The prostaglandin G/H synthase (PTGS/COX) system, ubiquitously expressed in mammalian tissues, exhibits dual catalytic activities (cyclooxygenase and peroxidase). It governs the conversion of arachidonic acid to prostaglandin precursors (Anamthathmakula and Winuthayanon, 2021). The constitutive COX-1 (PTGS1) maintains physiological homeostasis, whereas inducible COX-2 (PTGS2) demonstrates stimulus-responsive expression dynamics. Network pharmacology and molecular docking analyses indicate Marein potentially inhibits PTGS2 activity, thereby reducing pro-inflammatory prostaglandin production. Notably, COX-2 expression undergoes rapid upregulation (10–20-fold within 24 h) via NF-κB activation during inflammatory insults (Rao and Knaus, 2008), a process that Marein’s antioxidant properties may synergistically suppress.

As a non-receptor tyrosine kinase, SRC orchestrates cell survival and inflammatory/apoptotic signaling through PI3K/AKT and MAPK pathways. In CIRI pathophysiology, PI3K/AKT activation attenuates microglial hyperactivation, reduces pro-inflammatory cytokine release, and enhances neuronal survival (Li J. et al., 2024; Wang et al., 2012). Our molecular docking results revealed high binding affinity between Marein and SRC’s kinase domain, suggesting its potential to modulate PI3K/AKT signaling. This mechanism aligns with previous findings demonstrating PI3K/AKT inhibition reduces neuronal apoptosis and improves neurological deficit scores in CIRI models (Li et al., 2023).

Calcium ions (Ca^2+^), crucial secondary messengers, maintain a steep transmembrane gradient through voltage-gated channels, ATP-dependent pumps, and endoplasmic reticulum storage (Hu and Song, 2017). CIRI disrupts this equilibrium via membrane integrity loss, ATP depletion, and PKC-mediated activation of sodium-calcium exchangers (NCX) and voltage-dependent Ca^2+^ channes (Hong et al., 2023; Song and Yu, 2014). The resultant calcium overload exacerbates mitochondrial dysfunction and activates Ca^2+^-dependent proteases, culminating in irreversible cellular damage (Kristián and Siesjö, 1996). Pathway enrichment suggests Marein may mitigate calcium overload through TRP channel modulation (e.g., TRPV1) or calmodulin-dependent enzyme regulation, thereby preserving mitochondrial function and neuronal viability.

Despite providing valuable insights into the potential mechanisms of Marein in treating CIRI, this study has several limitations that should be acknowledged. First, network pharmacology-based target prediction relies on bioinformatics databases and computational algorithms, which may introduce inherent biases and inaccuracies. The identified targets, particularly PTGS2 and SRC, require further experimental validation to confirm their roles in the therapeutic effects of Marein. Second, molecular docking and molecular dynamics simulations, while useful for assessing binding interactions, have inherent limitations. The simulation time used in this study, though sufficient for preliminary observations, may not fully capture the long-term stability and dynamic behavior of Marein-target interactions, particularly within the calcium signaling pathway. Extending simulation durations and incorporating enhanced sampling techniques could improve the robustness of these findings. For future research, *in vivo* experiments are essential to validate the computational predictions. Cell-based assays can be employed to confirm the modulation of calcium signaling by Marein, while animal models of CIRI can provide further insights into its neuroprotective effects. Integrating multi-omics approaches, such as transcriptomics, may further elucidate the molecular mechanisms underlying Marein’s neuroprotective effects and identify novel therapeutic targets. By addressing these limitations and expanding experimental validation, future studies can provide a more comprehensive understanding of Marein’s potential as a therapeutic agent for CIRI.

Molecular docking and molecular dynamics simulation revealed the stable binding of Marein and SRC, indicating a high-affinity interaction. SRC, a non-receptor tyrosine kinase, plays a pivotal role in multiple cellular signaling pathways, including calcium signaling (Villalobo, 2023). Calcium signaling is critically involved in CIRI by modulating intracellular calcium homeostasis. Dysregulated calcium influx triggers mitochondrial impairment, apoptotic pathways, and oxidative stress, ultimately exacerbating neuronal injury and cell death (Wang et al., 2022). Activation of SRC kinase triggers the phosphorylation of calcium channel-associated proteins, enhancing channel activity and increasing intracellular calcium levels. The calcium overload subsequently activates calcium-dependent apoptotic pathways, ultimately leading to neuronal cell death (Christidis et al., 2022). The high-affinity interaction between Marein and SRC inhibits kinase activity, modulates calcium signaling, and suppresses calcium overload, attenuating CIRI through these coordinated mechanisms. IRI is a frequent complication after revascularization therapy for stroke, contributing to aggravated neuronal damage and the initiation of deleterious pathological cascades (Zhang H. et al., 2024). Given its detrimental impact on patient outcomes, mitigating IRI holds substantial significance in the therapeutic approach to stroke.

PTGS2, a key enzyme in prostaglandin synthesis, is involved in inflammatory responses and apoptosis (Zhuang et al., 2020). The attenuation of PTGS2 activity results in a diminution of inflammatory mediator production, thereby eliciting an anti-inflammatory effect (Jia et al., 2022). We propose that Marein may attenuate neuroinflammation in CIRI, through direct binding to PTGS2 and suppressing its catalytic function. Prostaglandins modulate intracellular calcium levels through G protein-coupled receptor (GPCR) signaling pathways (Jewell et al., 2011). Given that PTGS2 is a key enzyme in prostaglandin biosynthesis (Kamal et al., 2023), it may regulate calcium signaling through this metabolic cascade. Molecular docking and molecular dynamics simulations further demonstrated that Marein exhibits stable binding affinity with the epidermal growth factor receptor (EGFR). As a critical regulator of cell proliferation and survival, EGFR activation promotes oxidative stress and inflammatory responses (Wang et al., 2017), thereby exacerbating CIRI. The high-affinity interaction between Marein and EGFR likely inhibits its kinase activity, thereby attenuating downstream pro-inflammatory and apoptotic pathways. This newly identified mechanism complements Marein’s multi-target neuroprotective actions through SRC and PTGS2 inhibition, collectively providing comprehensive protection against CIRI pathogenesis.

Multi-target and multi-pathway drug delivery has emerged as a significant direction in modern drug development, offering substantial therapeutic benefits in disease treatment (Ryszkiewicz et al., 2023). The differential binding properties of Marein to SRC, PTGS2 and EGFR may reflect its selective actions on distinct targets. Inhibition of SRC, a central hub in signal transduction, could broadly influence multiple pathological pathways, while PTGS2 inhibition primarily targets inflammatory responses. Notably, EGFR inhibition further modulates oxidative stress and apoptotic pathways. This tripartite targeting capability positions Marein as a promising candidate for multi-faceted therapeutic intervention in CIRI. Furthermore, calcium signaling is crucial in neuroprotection (Zamponi et al., 2015). The binding of Marein to PTGS2 and SRC exerts distinct mechanisms of action within the calcium signaling pathway, which may further underpin the neuroprotective effect of Marein. When compared to the clinical standard Edaravone, an established neuroprotectant for ischemic stroke, Marein’s multi-target profile shows significant therapeutic promise. This polypharmacological approach appears to address the key limitations of single-pathway treatments like Edaravone, potentially providing more comprehensive neuroprotection throughout the ischemic cascade.

MMPBSA analysis was employed to evaluate the binding free energies of Marein and Edaravone with three core targets: EGFR, PTGS2, and SRC. Edaravone exhibited the most favorable binding with EGFR (−104.421 kJ/mol), followed by PTGS2 (−84.640 kJ/mol) and SRC (−36.433 kJ/mol). Despite stronger local interactions (van der Waals: up to −169.680 kJ/mol), Marein’s overall binding affinity was compromised by its higher polar solvation energy. These differences suggest that Marein may induce stronger local conformational effects, while Edaravone achieves tighter global binding. Together, these findings support the complementary mechanisms of these ligands in modulating signaling pathways relevant to oxidative stress and neuroinflammation, particularly in the context of CIRI.

This study demonstrates the protective role of Marein in HT22 cells subjected to OGD/R-induced injury. Marein’s protective profile is concentration-dependent: while lower concentrations (≤40 μM) are non-toxic, exposure to 80 μM leads to reduced viability, indicative of a cytotoxic threshold. This biphasic dose-response is characteristic of numerous natural flavonoids, including dihydromyricetin, which has also shown neuroprotective properties via Nrf2 pathway activation in similar models (Yu et al., 2021; Zhang et al., 2021). Marein significantly ameliorates OGD/R-induced cell death, consistent with prior reports involving natural antioxidants such as sevoflurane, which modulate PI3K/Akt signaling to preserve neuronal viability under ischemic conditions (Yu et al., 2021). The reduction in ROS levels observed with Marein treatment further supports its antioxidative function. Oxidative stress plays a central role in ischemia-reperfusion injury, promoting lipid peroxidation, protein modification, and DNA damage. Antioxidants that activate the Nrf2/HO-1 axis have been shown to confer protection in similar cellular models (Cheng et al., 2024; Qi et al., 2024). Although this study provides mechanistic insights into Marein’s neuroprotective effects using *in vitro* models and computational predictions, several limitations remain. The absence of *in vivo* validation and calcium signaling assays should be addressed in future work. Given that calcium signaling emerged as a potentially relevant pathway, further studies involving calcium influx detection and calcium channel modulators are needed to better define the underlying mechanisms. Additionally, experiments in animal models of cerebral ischemia-reperfusion would help to confirm the relevance of the identified targets *in vivo*.

## 5 Conclusion

This study primarily employed a network pharmacology-based approach, complemented by molecular docking and molecular dynamics simulations, to explore the potential mechanisms of Marein in the treatment of cerebral CIRI. Computational analyses identified SRC and PTGS2 as key targets, suggesting that Marein may exert neuroprotective effects by modulating calcium signaling and inflammatory pathways. Molecular docking and dynamic simulations further demonstrated stable binding of Marein to these targets, providing molecular-level support for its predicted bioactivity. To validate these predictions, preliminary *in vitro* experiments were conducted using HT22 cells subjected to OGD/R. Marein treatment significantly improved cell viability and reduced intracellular ROS levels, consistent with the proposed antioxidative and cytoprotective mechanisms. These findings highlight Marein’s potential as a multi-target agent in mitigating ischemia-induced neuronal injury and lay a theoretical foundation for further preclinical investigations.

## Data Availability

The original contributions presented in the study are included in the article/Supplementary Material.

## Supplementary material

SUPPLEMENTARY FIGURE S1 Conformational changes of the Marein-PTGS2 complex over 100 ns molecular dynamics simulation. Snapshots at 0 ns, 20 ns, 40 ns, 60 ns, 80 ns, and 100 ns show structural evolution of PTGS2 (colored cartoon) with bound Marein (spheres).

SUPPLEMENTARY FIGURE S2 Conformational changes of the Marein - Src complex over molecular dynamics simulation. Snapshots at 0 ns, 20 ns, 40 ns, 60 ns, 100 ns, and 180 ns show structural evolution of Src (colored cartoon) with bound Marein (spheres).

SUPPLEMENTARY FIGURE S3 Conformational dynamics of the Marein - EGFR complex over a 100 - ns molecular dynamics simulation. Snapshots at 0 ns, 20 ns, 40 ns, 60 ns, 80 ns, and 100 ns illustrate structural changes of EGFR (colored cartoon) with bound Marein (spheres).

SUPPLEMENTARY FIGURE S4 Conformational changes of the Edaravone - PTGS2 complex over a 100 - ns molecular dynamics simulation. Snapshots at 0 ns, 20 ns, 40 ns, 60 ns, 80 ns, and 100 ns show structural evolution of PTGS2 (colored cartoon) with bound Edaravone.

SUPPLEMENTARY FIGURE S5 Conformational dynamics of the Edaravone - Src complex over a 100 - ns molecular dynamics simulation. Snapshots at 0 ns, 20 ns, 40 ns, 60 ns, 80 ns, and 100 ns show structural changes of Src (colored cartoon) with bound Edaravone.

SUPPLEMENTARY FIGURE S6 Conformational changes of the Edaravone - EGFR complex over a 100 - ns molecular dynamics simulation. Snapshots at 0 ns, 20 ns, 40 ns, 60 ns, 80 ns, and 100 ns show structural evolution of EGFR (colored cartoon) with bound Edaravone.

SUPPLEMENTARY FIGURE S7 Full - membrane Western blots showing protein expression levels. **(A)** PTGS2 and β - actin; **(B)** P - Src and β - actin; **(C)** Src and β - actin. Treatments: OGD/R (oxygen - glucose deprivation/reperfusion) and Marein (20 μM).

SUPPLEMENTARY VIDEO 1 Molecular dynamics simulation trajectories (100 ns) of six protein–ligand complexes: **(A)** Marein–PTGS2, **(B)** Marein–Src, **(C)** Marein–EGFR, **(D)** Edaravone–PTGS2, **(E)** Edaravone–Src, and **(F)** Edaravone–EGFR.

## Abbreviations

CIRI, Cerebral Ischemia-Reperfusion Injury; GO, Gene Ontology; KEGG, Kyoto Encyclopedia of Genes and Genomes; CC, Cell components; MF, Molecular functions; BP, Biological processes; PPI, Protein-Protein Interaction; OGD/R, oxygen - glucose deprivation/reperfusion.
