# Supplementary material for: Elucidating multifaceted targets of Marein in cerebral ischemia-reperfusion injury
Source: Front Chem. 2025 Oct 9;13:1651873. doi: 10.3389/fchem.2025.1651873 (PMC12547697; doi:10.3389/fchem.2025.1651873)

Elucidating Marein's Neuroprotection in Cerebral Ischemia-Reperfusion Injury via Multi - Omics Methods

Weidan Luo[1,2†], Jian Wang[3,4†], Ying Fan[1], Meng Yuan[1], Wanying Xie[1], Yang Su[1], Xingchun Wang[5], Yi Zhong[1], Yibo Zhang[1], Jiaxin Zhan[1], Xuan Mao[1], Xinyao Huang[1], Junxi Long[1], Xinrui Wang[1], TingTing Tang[1], Xingxia Wang[1*]

[1] Department of Neurology, The Affiliated Hospital, Southwest Medical University, Luzhou, Sichuan, China.

[2] Department of General Surgery (Vascular Surgery), The Affiliated Hospital, Southwest Medical University, Luzhou, Sichuan, China.

[3] Department of Comprehensive (VIP) Inpatient Ward, Sichuan Clinical Research Center for Cancer,Sichuan Cancer Hospital & Institute, Sichuan Cancer Center, University of Electronic Science and Technology of China, Chengdu, China.

[4] Department of Oncology, Luzhou People's Hospital, Luzhou, Sichuan, China.

[5] The Affiliated Stomatological Hospital of Southwest Medical University, Southwest Medical University Luzhou, Sichuan, China.

[*] Corresponding authors: Dr. Xingxia Wang; e-mail: xingxiawang888@swmu.edu.cn

[†] These authors contributed equally to this work.

Figure S1

Marein-PTGS2

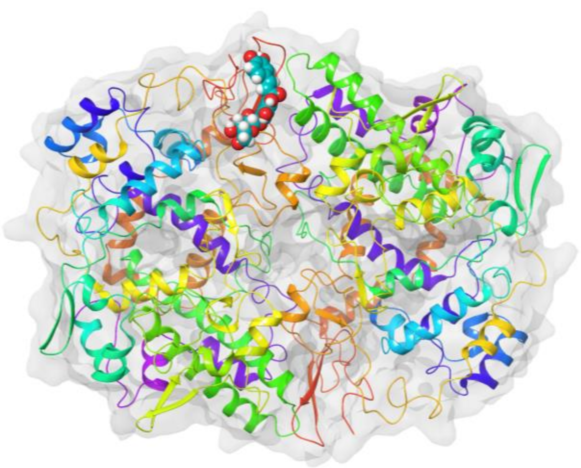

0ns

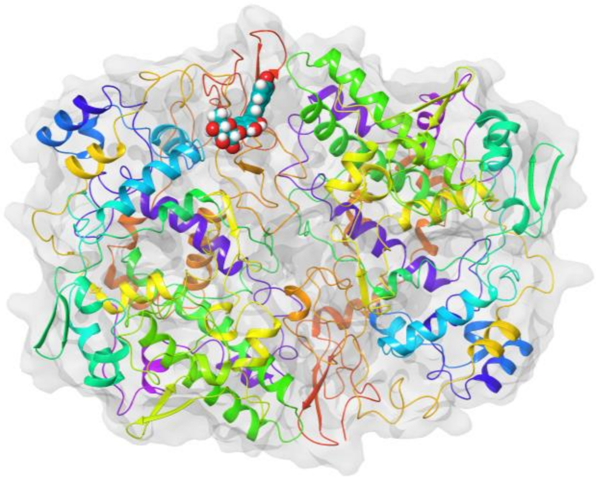

20ns

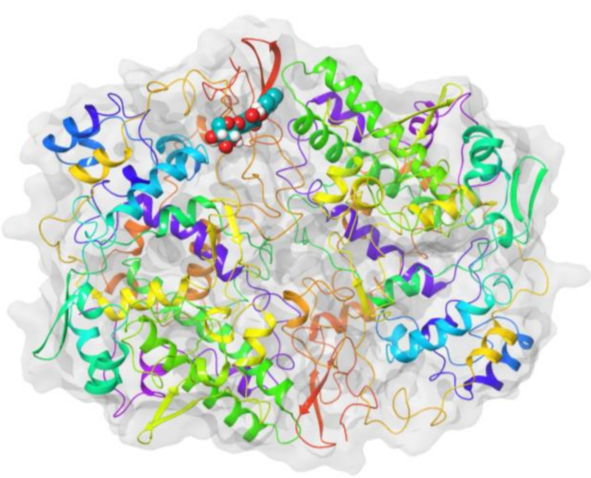

40ns

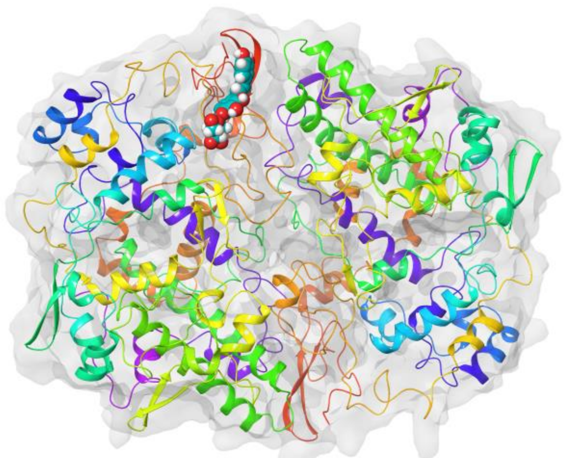

60ns

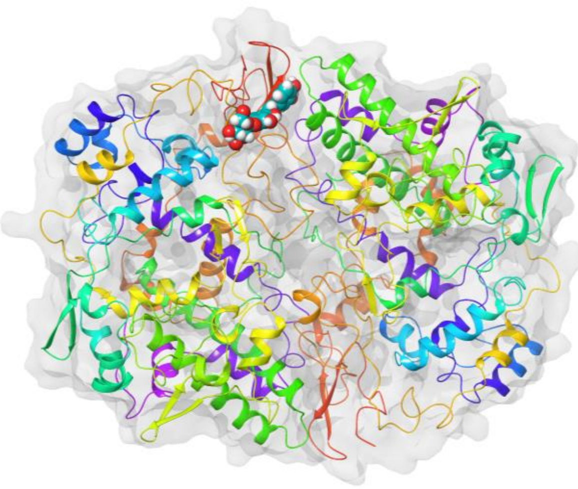

80ns

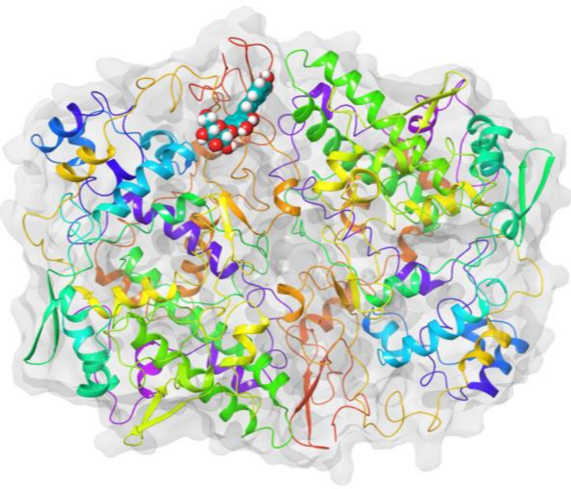

100ns

Figure S2

Marein-Src

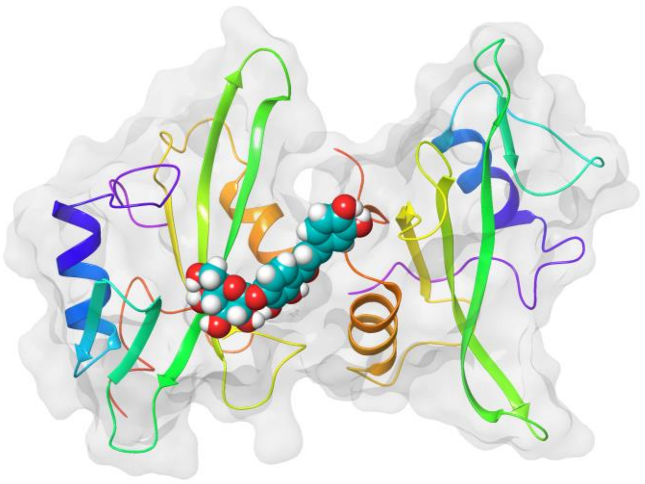

0ns

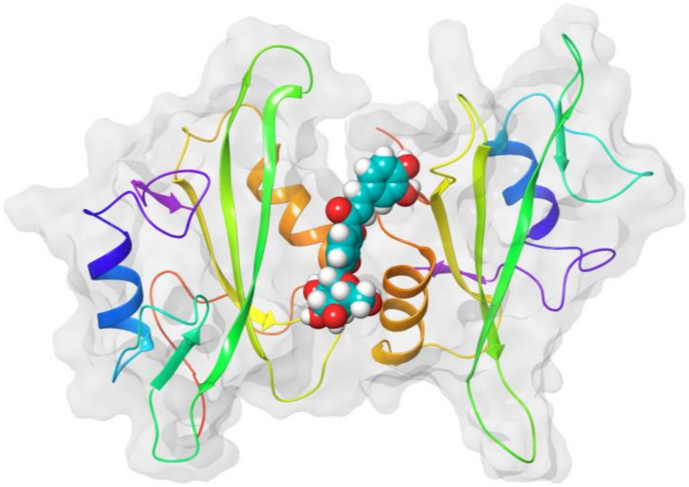

20ns

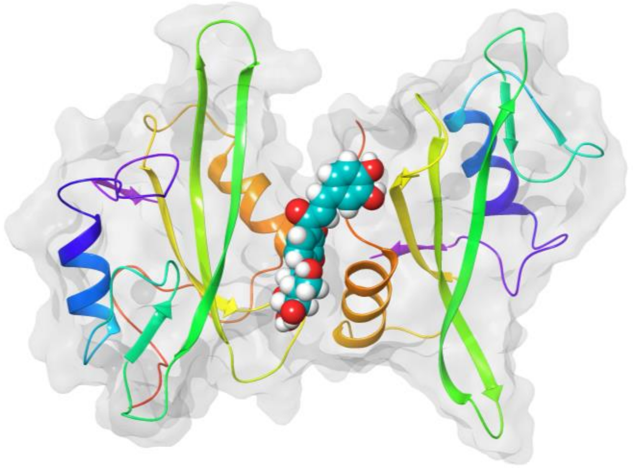

40ns

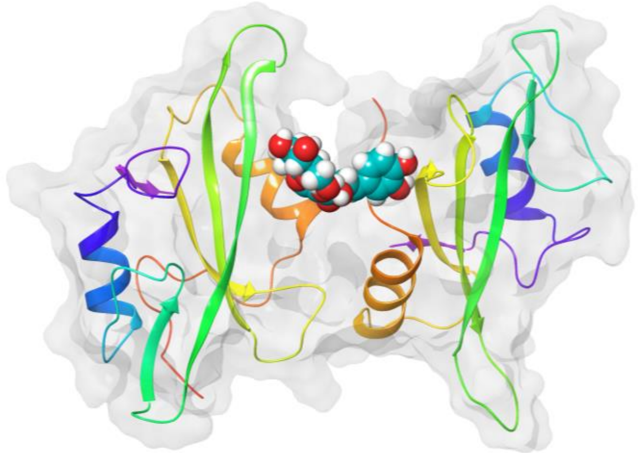

60ns

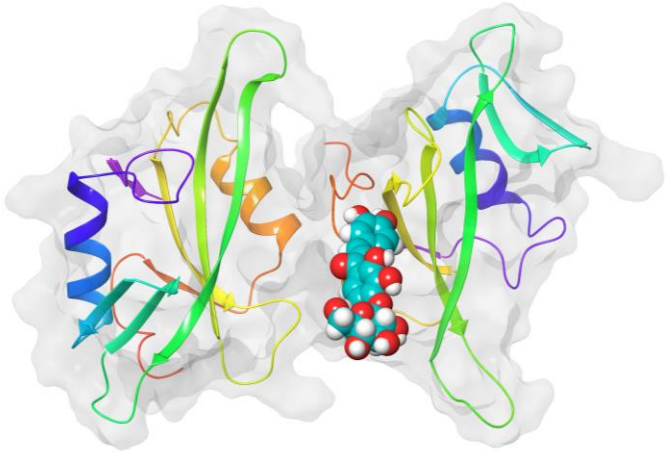

100ns

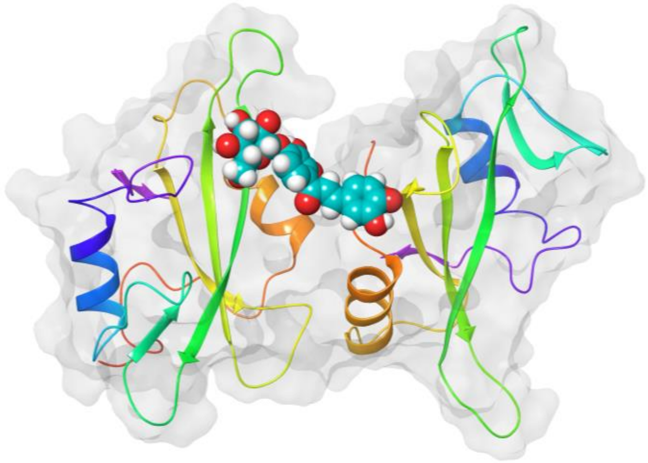

180ns

Figure S3

Marein-EGFR

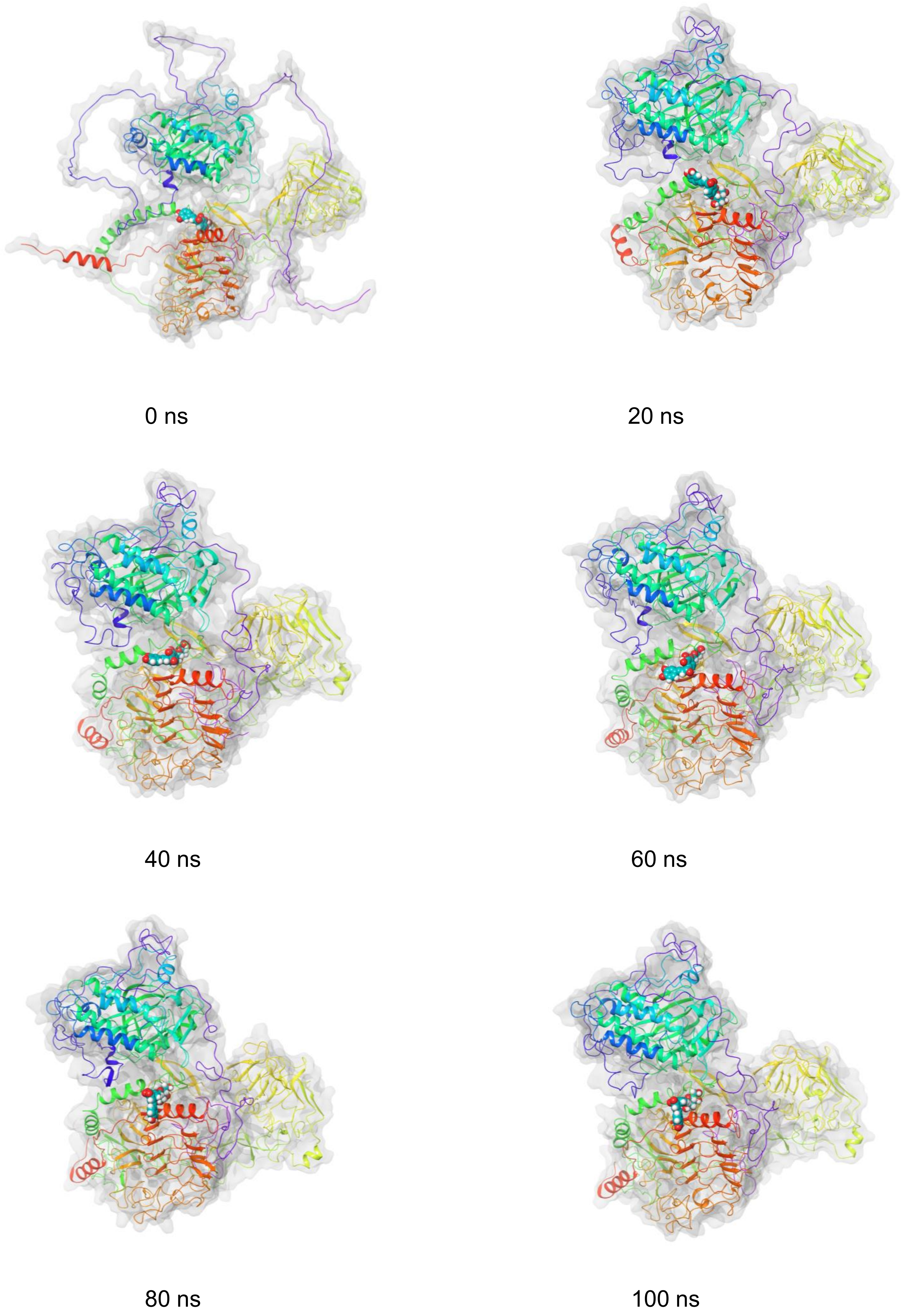

Figure S4

Edaravone-PTGS2

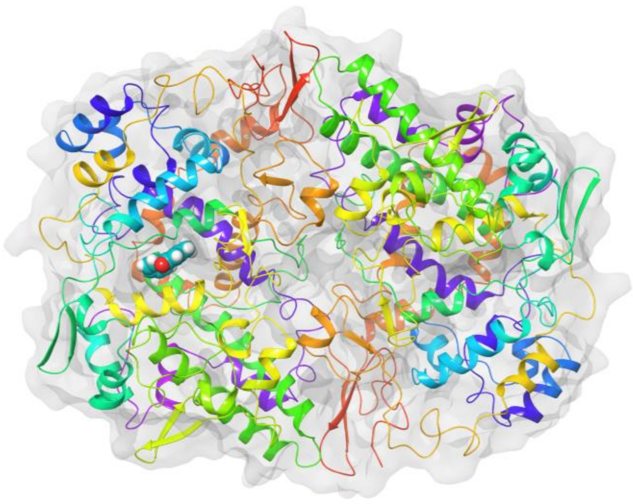

0

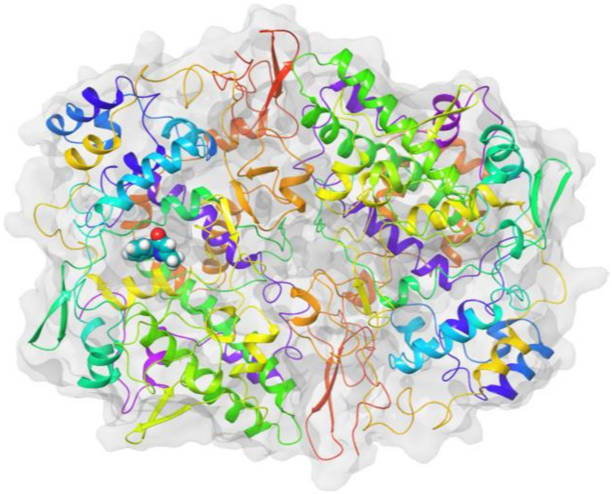

20 ns

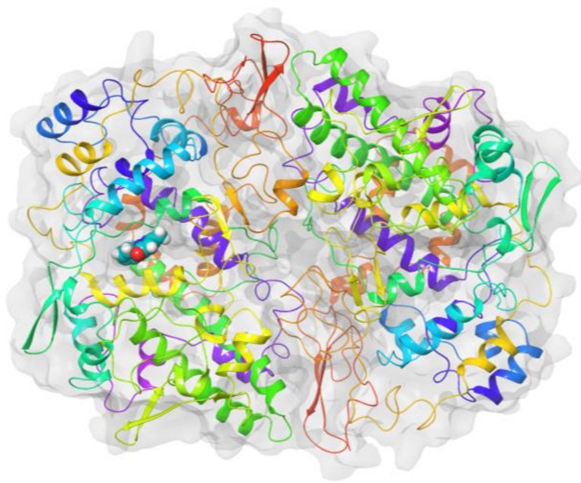

40 ns

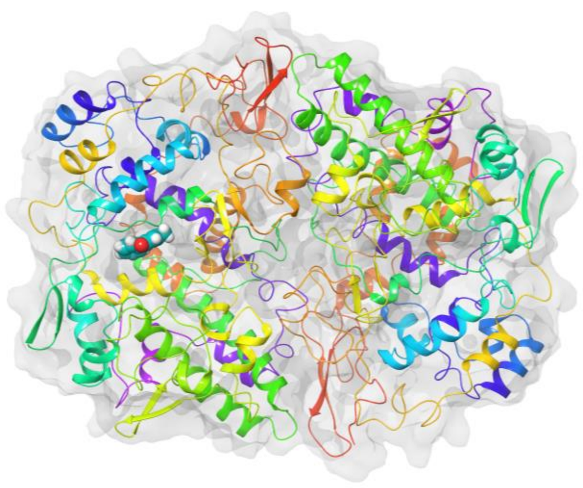

60 ns

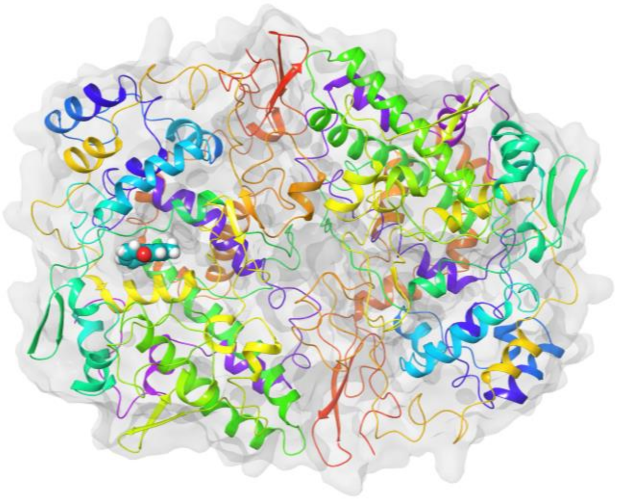

80 ns

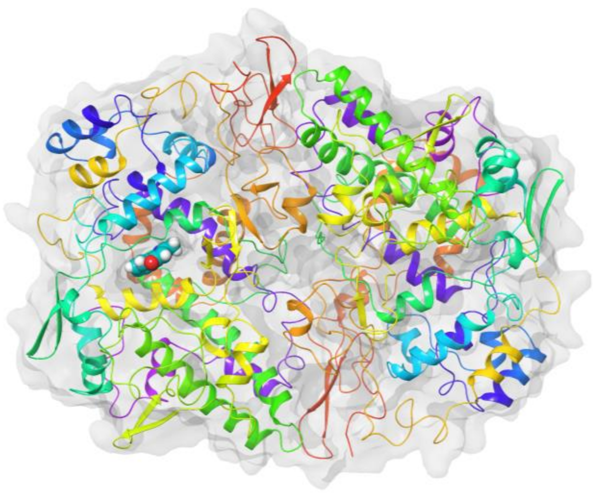

100 ns

Figure S5

Edaravone-Src

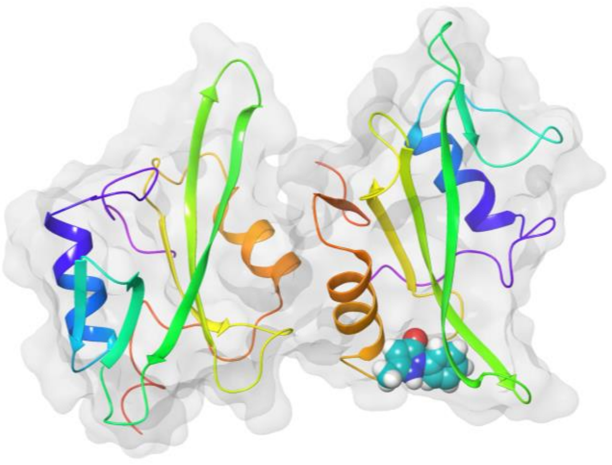

0 ns

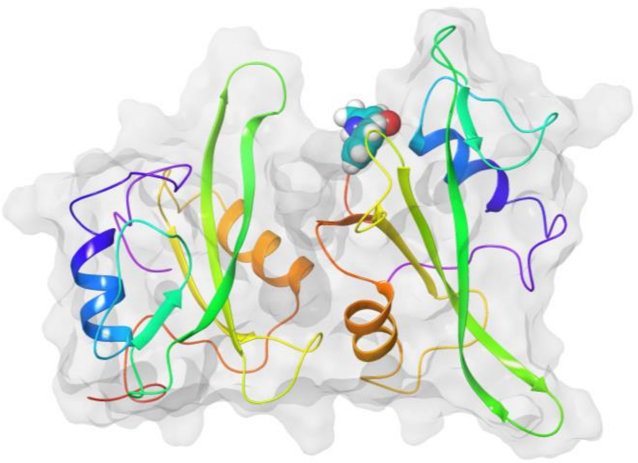

20 ns

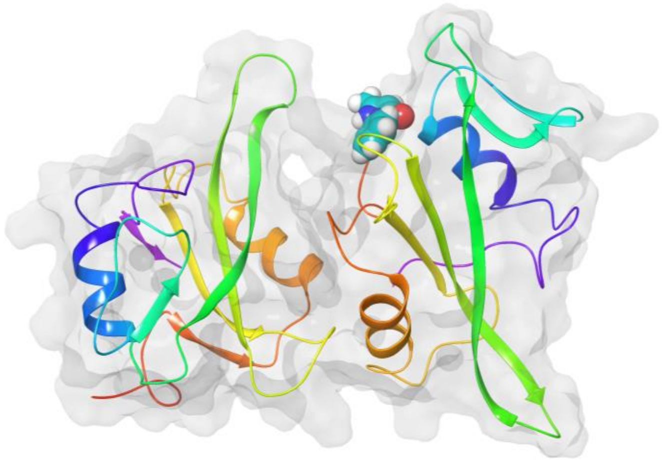

40 ns

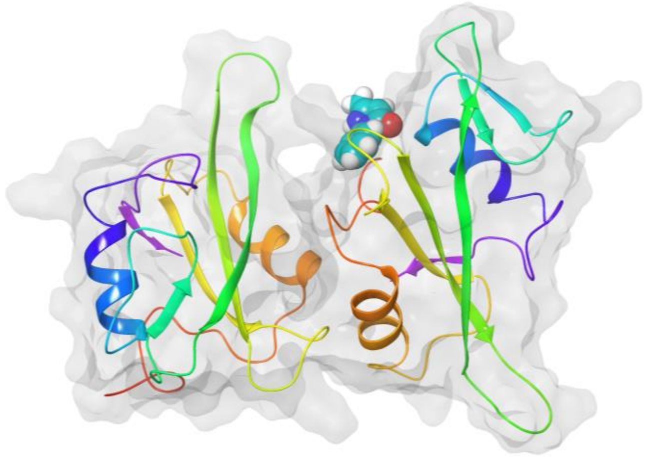

60 ns

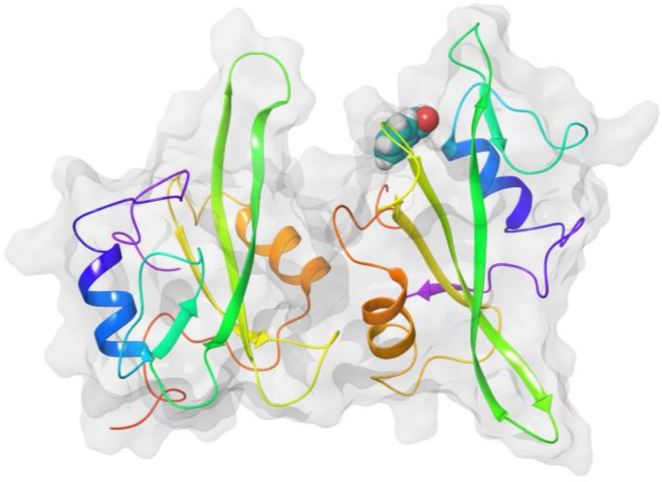

80 ns

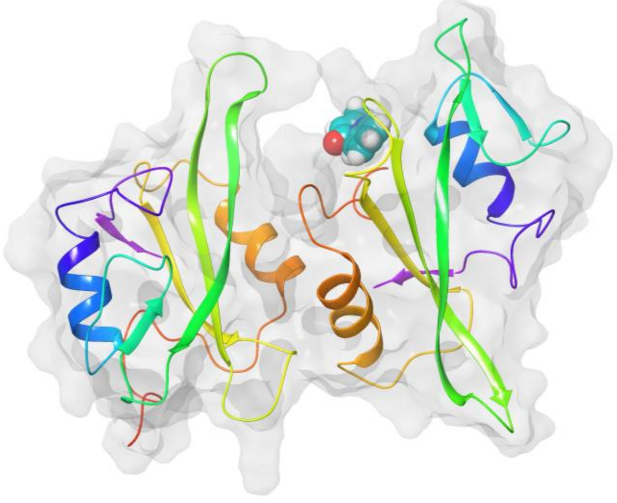

100 ns

Figure S6

Edaravone-EGFR

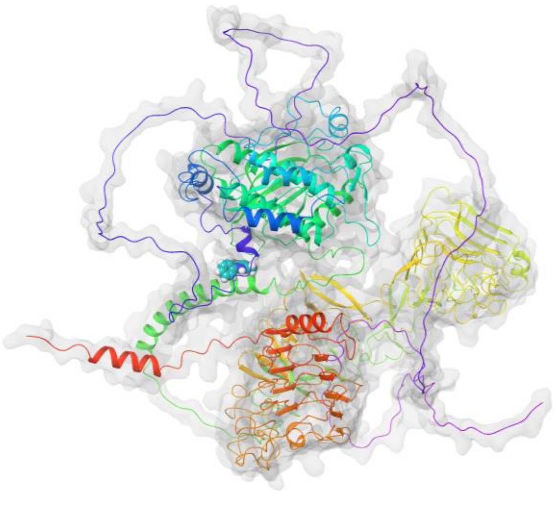

0ns

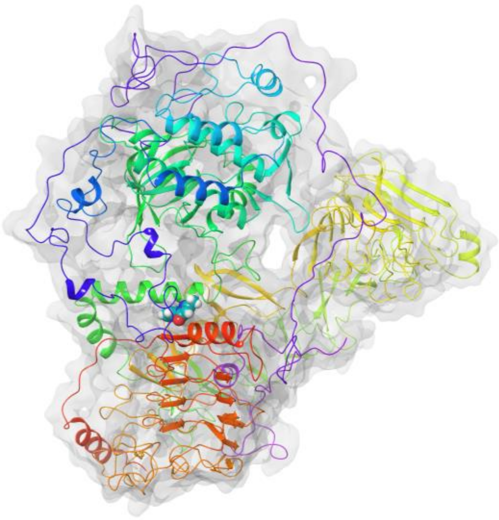

20ns

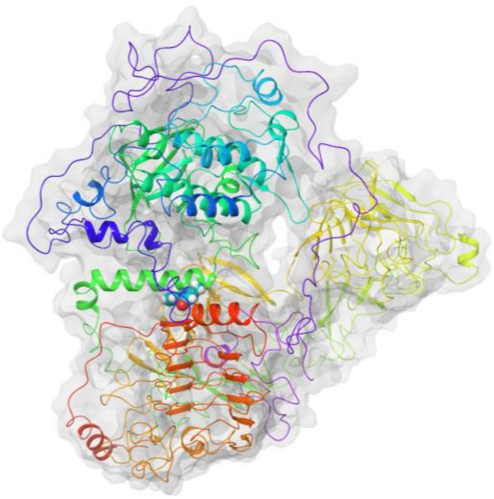

40ns

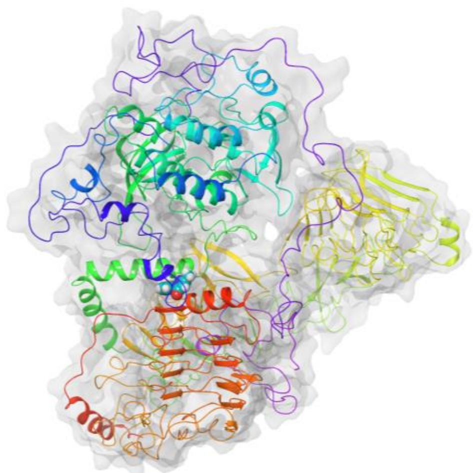

60ns

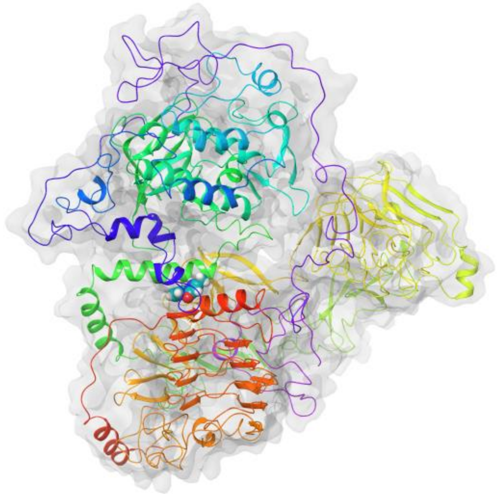

80ns

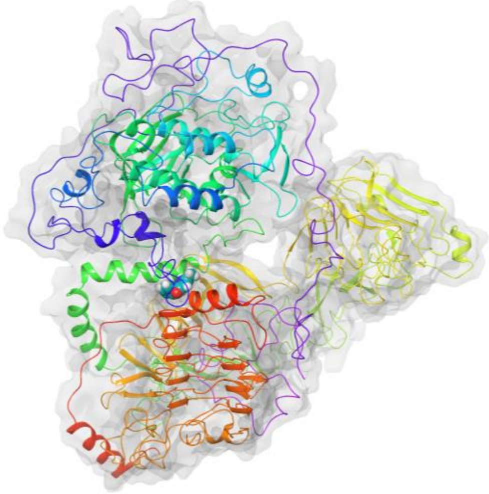

100ns

Figure S7

A

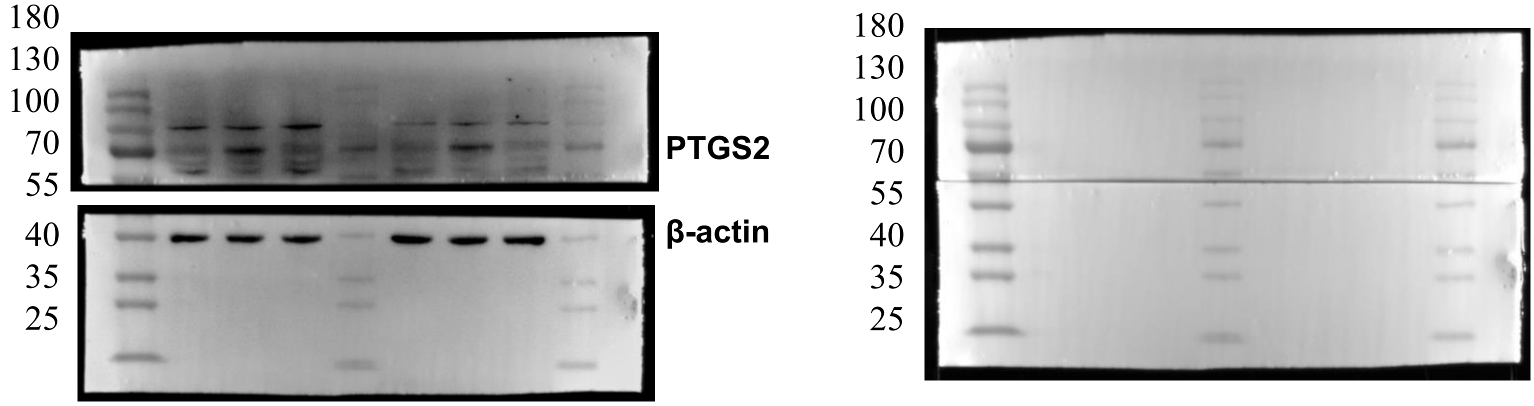

B

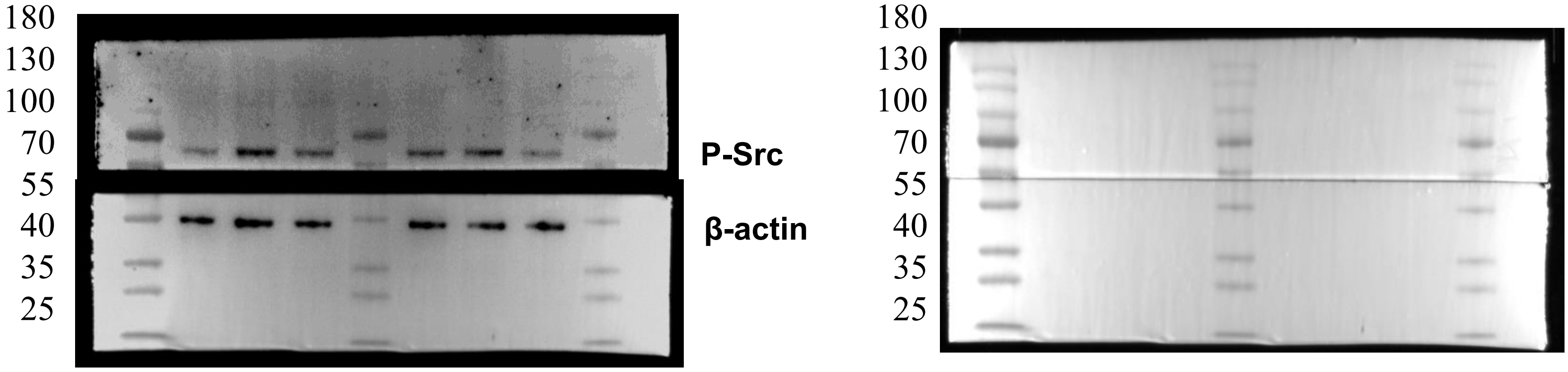

C

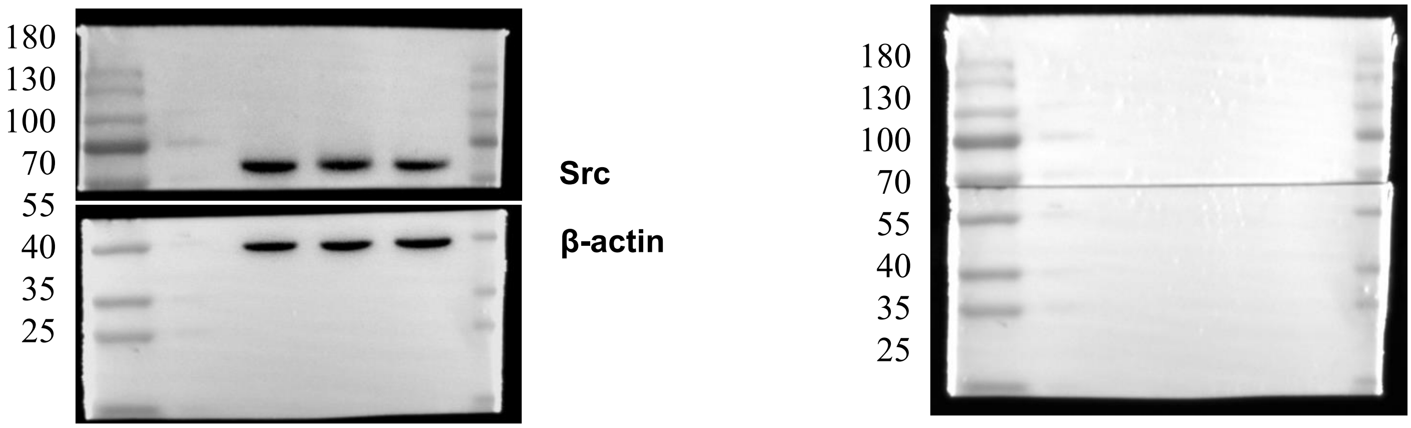

Supplement: Supplementary file 2 [file Image1.pdf]
